# Effectiveness and safety of biological and target synthetic drugs treatment for psoriatic arthritis: a systematic review with network meta-analysis

**DOI:** 10.1186/s42358-024-00361-3

**Published:** 2024-03-21

**Authors:** Thais Montezuma, Livia Fernandes Probst, Matheus Oliveira Almeida

**Affiliations:** 1 Health Technology Assessment Unit, Hospital Alemão Oswaldo Cruz, Rua Treze de Maio, 1815 - Bela Vista, São Paulo, SP 01323-020 Brazil; 2 Management and Collective Health, State University of Campinas, Campinas, Brazil

**Keywords:** Psoriatic arthritis, Biological medicines, Systematic review, Network meta-analysis

## Abstract

**Background:**

Psoriatic arthritis (PA) is a chronic inflammatory systemic arthritis that can result in loss of functional capacity and joint deformation. This systematic review assessed the effectiveness and safety of biological and target synthetic drugs for treating PA.

**Methods:**

We searched for randomized clinical trials (RCTs) that evaluated the use of Adalimumab, Etanercept, Infliximab, Golimumab, Secukinumab, Certolizumab Pegol and Tofacitinib in the main general databases and clinical trial registers databases. The primary outcomes were ACR 50, PsARC, and serious adverse events. Two independent reviewers performed study selection and data extraction. Network meta-analyses were conducted using a random effects model and frequentist approach. The CINeMA software was used to assess the certainty of evidence.

**Results:**

We included 33 RCTs (n = 11,034). The results from the network meta-analysis for the ACR 50 at 6-months follow-up showed that all drugs were superior to placebo, with Secukinumab (high certainty of evidence), Infliximab (very low certainty of evidence) and Adalimumab (high certainty of evidence) ranking the highest. Regarding the PsARC (at 6-months follow-up), all drugs, except for Golimumab (very low certainty of evidence), were superior to placebo, with Etanercept (low certainty of evidence), Infliximab (low certainty of evidence) and Certolizumab Pegol (low certainty of evidence) being the most effective drugs. There were no significant differences in the risk of serious adverse events between the drugs and placebo. Golimumab (very low certainty of evidence), Secukinumab (low certainty of evidence), and Adalimumab (very low certainty of evidence) ranked the highest for safety.

**Conclusions:**

In conclusion, based on the balance between efficacy and safety, Secukinumab and Adalimumab may be the preferred options among the evaluated drugs for treating patients with PsA. However, caution is necessary when interpreting the safety findings, as they are supported by evidence of low to very low certainty. Consequently, the balance between benefits and potential risks may change as new safety evaluation studies become available.

**Protocol registration:**

PROSPERO: CRD42022315577

**Supplementary Information:**

The online version contains supplementary material available at 10.1186/s42358-024-00361-3.

## Background

Psoriatic arthritis is a chronic and systemic inflammatory condition that affects both the peripheral joints and axial skeleton and is commonly associated with psoriasis. It is typically characterized by enthesitis, dactylitis, and nail and skin involvement [1]. The global prevalence of psoriatic arthritis ranges from 0.3 to 1% of the population, and its incidence ranges from 0.01 to 5 cases per 100,000 individuals [2]. In patients with psoriasis, psoriatic arthritis occurs in up to 30% of cases [2, 3]. It is estimated that psoriatic arthritis is the second most common spondylarthritis in Brazil, with a prevalence rate of 13.7% [4].

The treatment of psoriatic arthritis aims to prevent loss of functional capacity and joint deformation, as well as to control symptoms and disease activity, ultimately improving quality of life [5]. The Brazilian Clinical Protocol and Therapeutic Guideline for Psoriatic Arthritis recommends a variety of drug therapies, including non-steroidal anti-inflammatory drugs and corticosteroids, as well as synthetic disease-modifying antirheumatic drugs (csDMARD-IR), biological disease-modifying antirheumatic drugs (bDMARDs) and specific target synthetic drugs (MMCDsae) [6].

Clinical guidelines recommend the use of bDMARDs and MMCDsae when disease activity persists despite the use of two therapeutic regimens with DMARDs for at least six months [6, 7]. The bDMARDs available in the Brazilian Unified Health System (in portuguese, Sistema Único de Sáude—SUS) are Adalimumab, Etanercept, Golimumab, Infliximab, Secukinumab, Certolizumab Pegol, whereas the MMCDsae is Tofacitinib [6]. These drugs are provided by the Specialized Component of Pharmaceutical Assistance of SUS, and their provision must adhere to the Clinical Protocols and Therapeutic Guidelines of the Brazilian Ministry of Health, which include parameters such as diagnosis, treatment indication, patient inclusion and exclusion criteria, therapeutic regimens, monitoring, and follow-up.

Previous systematic reviews have indicated that bDMARDs and MMCDsae can alter the course and activity of psoriatic arthritis [8–11]. However, some of these reviews may have had methodological limitations and/or evaluated drugs not available in Brazil. This review aims to exclusively evaluate the effectiveness and safety of bDMARDs and MMCDsae indicated in Brazil, making it the first of its kind. Additionally, we aim to rank these treatments according to their efficacy and safety.

## Methods

This systematic review followed the recommendations of the Cochrane Handbook for Systematic Reviews of Interventions and was reported in accordance with the PRISMA-NMA (Reporting Items for Systematic Review and Meta-Analyses) [12–14]. The protocol for this systematic review was registered in the International Prospective Register of Systematic Reviews (PROSPERO) database and assigned the number CRD42022315577.

### Eligibility criteria

We included studies that evaluated adult patients (≥18 years old) diagnosed with psoriatic arthritis according to internationally validated and established criteria, with no restrictions on the severity of psoriatic arthritis at baseline. We only included studies that evaluated a population with conditions other than psoriatic arthritis (example: another rheumatic disease and psoriatic arthritis; or psoriatic plaque and psoriatic arthritis) if they reported the data separately or if the majority of participants (>75%) had psoriatic arthritis.

We considered eligible studies that assessed the use of biological drugs (Adalimumab, Etanercept, Infliximab, Golimumab, Secukinumab, Certolizumab pegol) as well as synthetic target-specific drugs (tofacitinib) in the treatment of psoriatic arthritis. The use of these drugs could be isolated or in combination with methotrexate (MTX). Only doses recommended in the Brazilian Clinical Protocol and Therapeutic Guidelines for Psoriatic Arthritis of the Ministry of Health were included (Adalimumab—40 mg; Etanercept—50 mg; Infliximab—5 mg/kg; Golimumab—50 mg; Secukinumab 150 and 300 mg; Certolizumab pegol—200 or 400 mg; Tofacitinib—5 mg) [6]. If studies reported multiple approved doses of the same drug, they were grouped into a single treatment arm, in accordance with the *Cochrane Handbook* [12]. We include studies that compared the biological drugs cited above with each other, as well as with synthetic disease-modifying drugs (example: MTX), placebo and with no treatment.

The choice of assessed outcomes was based on the updated list of main outcome domains for Psoriatic Arthritis from the Outcome Measures in Rheumatology (OMERACT) initiative [15]. We also looked for the expertise of a rheumatologist to define the relevant outcomes of interest. These outcomes were categorized into 7 domains: (1) disease response; (2) function; (3) disease activity; (4) pain; (5) quality of life; (6) skin disease; (7) adverse events. The primary outcomes were: Disease response (ACR 50); Psoriatic Arthritis Response Criteria (PsARC); serious adverse events. Secondary outcomes included: Function (Health Assessment Questionnaire Disability Index—HAQ-DI); disease response (example: Disease Activity in Psoriatic Arthritis Score—DAPSA; CDAI; DAS-28; Minimal Disease Activity—MDA); pain; quality of life (SF-36 or Psoriatic Arthritis Quality of Life—PSORIQOL); skin disease (PASI Score); total adverse events; adherence and discontinuation.

We only included randomized or quasi-randomized clinical trials (RCTs). There was no restriction regarding the year or language of publication of the studies. Transition studies, switching studies and interchangeability studies were not considered. If a study had multiple parts, we considered only the results of the first period (direct comparison between the interventions of interest).

### Information sources

In February 2022, we conducted searches for RCTs in the following electronic databases: The Cochrane Central Register of Controlled Trials (CENTRAL), MEDLINE (via PubMed), EMBASE (via OvidSP), Latin American and Caribbean Health Science (LILACS). Additionally, we searched for unpublished or ongoing studies in the following clinical trial registry databases: EU Clinical TrialRegister (https://www.clinicaltrialsregister.eu), International Clinical Trials Registry Platform-World Health Organization (http://apps.who.int/trialsearch/), and Clinicaltrials (https://clinicaltrials.gov/). We also manually searched the reference lists of potential studies to be included and previously published systematic reviews.

### Search strategy

The search strategies were created using controlled vocabulary terms specific to each database, along with free terms relevant to the research question. Terms related to the condition under investigation (psoriatic arthritis) were combined with terms pertaining to all biological drugs of interest in the present review and terms relating to the study design (randomized clinical trials). The search was customized for each database individually (Additional file—Table S1)

### Selection process

Two reviewers initially screened titles and abstracts independently. The full text of every potentially relevant study was then obtained to determine its eligibility for inclusion. The reasons for exclusion were recorded. Any disagreements were resolved through discussion or with the assistance of a third reviewer as an arbitrator. We identified and excluded duplicates, as well as compiled multiple reports of the same study. The study selection process was performed in the Covidence software [16].

### Data extraction process

We extracted the data using a standardized form designed for this review in the Covidence software [16]. Two reviewers performed the data extraction independently, and disagreements were resolved by consensus. When necessary, a third reviewer resolved disagreements.

The following data were extracted: publication data (author, year of publication, language); characteristics of the studies (study design, setting, number of centers, sample size, size of each treatment arm, study duration, conflict of interest and funding); characteristics of the participants (gender, age, duration of illness); characteristics of the intervention (route of administration, dosage, frequency of treatment, adjuvant therapy); evaluated outcomes and the respective time points.

If studies presented multiple measurements for the same outcome, we adopted the following order of preference:

1. HAQ-DI score, followed by the proportion of participants who achieved a minimal clinically important difference of at least 0.22.
2. Disease activity: DAPSA, followed by DAS28 and CDAI or MDA.
3. Pain: visual analogue pain scale, followed by numeric pain scale.
4. Quality of Life: PSORIQOL, followed by SF-36.
5. Skin disease: PASI score, followed by the 75% reduction ratio (PASI 75).

For quantitative outcome data, we extracted measures of central tendency (preferably mean values) and variability (preferably standard deviation values) from each treatment group for continuous outcomes. If standard deviations (SD) were not reported, we calculated them from other statistical measures (such as standard error, confidence intervals), as recommended by the *Cochrane Handbook* [12]. If we were unable to calculate the SD, we performed imputation (for example, using the SD of the baseline treatment arms or others included in the meta-analysis).

We prioritized extracting the mean standard deviation for each follow-up time in each treatment arm for continuous data. If this unavailable, we extracted the change from baseline data. For dichotomous outcomes, we collected the number of events and number of participants in each treatment group.

We extracted data for specific time points, including 6 months (or closer); 12 months (or closer); 24 months (or closer); 36 months (or closer) and so on (if data were available). When available, we preferred to extract data based on intention-to-treat analysis.

### Assessment of risk of bias in studies

We assessed the risk of bias of RCT at the outcome level using the Cochrane Risk of Bias tool 2.0 (RoB 2) [17]. We classified the RCTs as low risk of bias (if all domains were rated as low risk), some concerns (if at least one domain was rated as some concerns and no domains were rated as high risk), and high risk of bias (if at least one domain was rated as high risk or if multiple domains were rated as some concerns).

### Treatment effect measures

We presented the results using mean difference (MD) with the 95% confidence interval (95% CI) for continuous outcomes assessed by the same scale. When outcomes were assessed using different scales, we presented the results as standardized mean difference (SMD) and 95% CI. Dichotomous outcomes were presented as risk ratio (RR) with the 95% CI.

For each treatment, we estimated the ranking probabilities of being in each possible ranking for all outcomes. The treatment hierarchy was presented using the *P-score*, which ranges from 0 to 1 and represents the average degree of certainty for a treatment to be superior to the others in the network meta-analysis (when using the frequentist approach) [18].

### Evaluation of transitivity

To evaluate the transitivity assumption, we compared the distribution of potential effect modifiers (age; gender, weight, and duration of symptoms) across the different pairwise comparisons to ensure there were no substantial differences. We also assessed the similarity of control groups in treatment comparisons.

### Methods of synthesis

When there was enough homogeneous data available, a meta-analysis was conducted for each intervention pair being compared. The random effects model was used to perform the analysis and calculate the pooled treatment effect and corresponding 95% CI. The data was grouped based on similarities in drugs and comparators, as well as the time points evaluated (e.g. 6 months or the closest follow-up period; 12 months or the closest follow-up period; and so on if data was available). Pairwise meta-analyses were performed using STATA 14 software. Whenever possible, network meta-analyses (NMA) were conducted using a random effects model and a frequentist approach for all outcomes and comparisons, in order to estimate relative effects for all possible comparisons between any pair of treatments. The software used for conducting the network meta-analyses was NMAstudio [19].

To assess heterogeneity within each pairwise, we visually inspected the similarity of point estimates and overlapping confidence intervals and used the Chi² test and I² measure. The evaluation of the statistical heterogeneity of the complete network was based on Tau². We evaluated inconsistency in the network´s results for primary outcomes both locally using the side-splitting method and globally.

### Assessment of publication bias

We performed a publication bias analysis through visual inspection of the funnel plot, but only if more than 10 included studies were included.

### Additional analysis

We planned sensitivity analyses for the primary outcomes when sufficient data are available, considering the following characteristics: sample size (inclusion of only RCTs with a sample size of at least 100 participants per treatment arm); industry funding (only RCT without funding were included); age of participants (inclusion of RCTs with participants aged 65 years or older) and risk of bias (inclusion of RCTs with low risk of bias). Furthermore, in addition to the sensitivity analyses, we planned to analyze the outcomes of interest considering only studies conducted in Brazil within the context of SUS.

### Certainty of evidence assessment

We evaluated the certainty of evidence for each primary outcome in each comparison using the CINeMA approach. This approach is based on six domains: within-study bias; reporting bias, indirectness (includes transitivity); imprecision; heterogeneity and incoherence. The overall assessment of the certainty of the evidence for each outcome was classified as high, moderate, low, or very low. Additionally, we created a ‘Summary of Findings’ tables to provide a concise summary of the primary outcomes ACR50 and serious adverse events.

## Results

### Studies selection

**Fig. 1:**
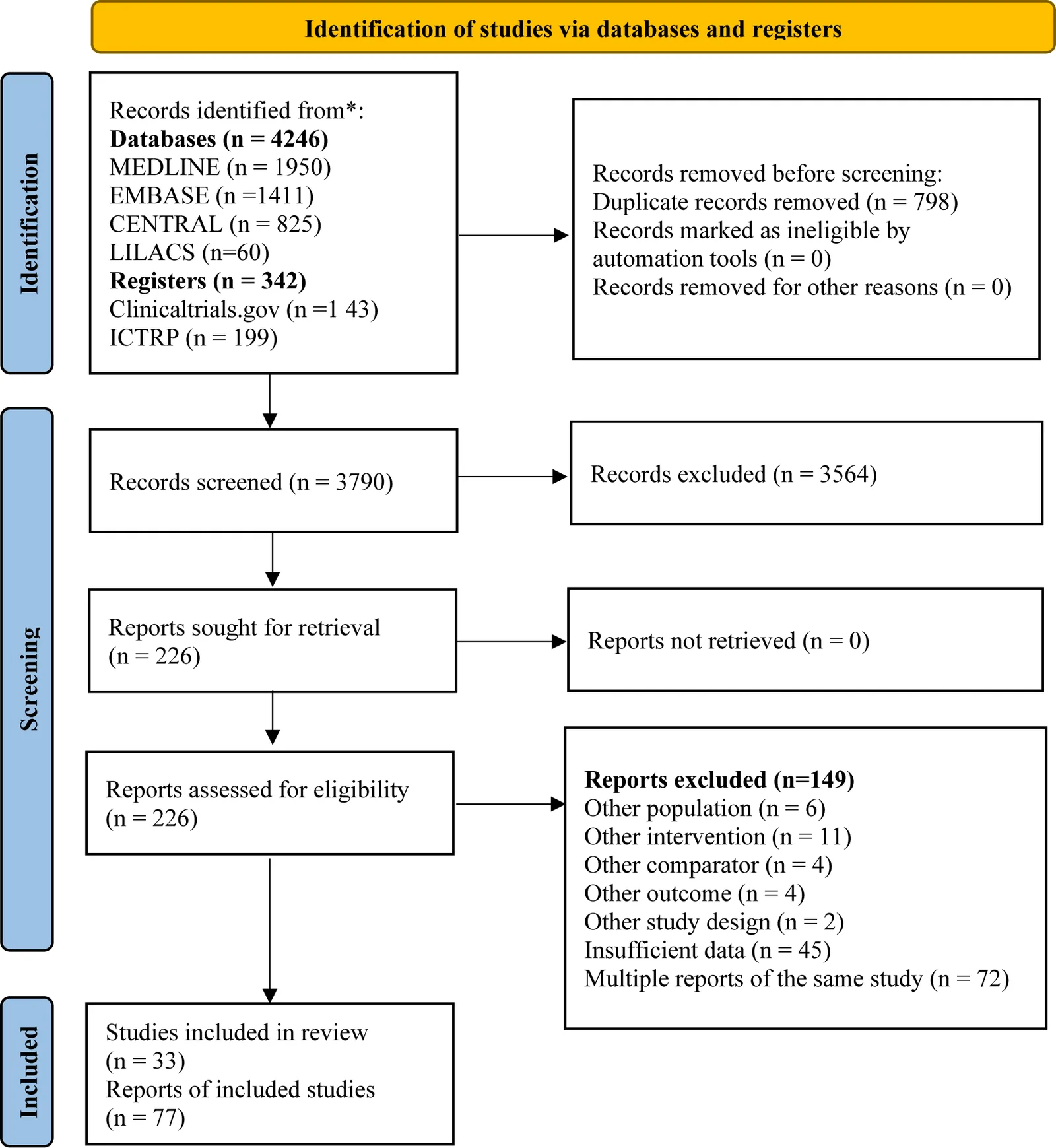
Flow diagram of study selection process

The search for RCTs in databases identified a total of 4568 references, comprising of 4246 publications and 342 clinical trials registers. After removing duplicates, 3790 underwent screening of titles and abstracts, resulting in the selection of 226 references for full text assessment. Ultimately, the review included 33 studies (77 publications). Figure 1 shows the flowchart of the selection process, and the excluded studies from the full-text eligibility analysis are available in Additional file—Table S2.

### Presentation of the network structure

**Fig. 2:**
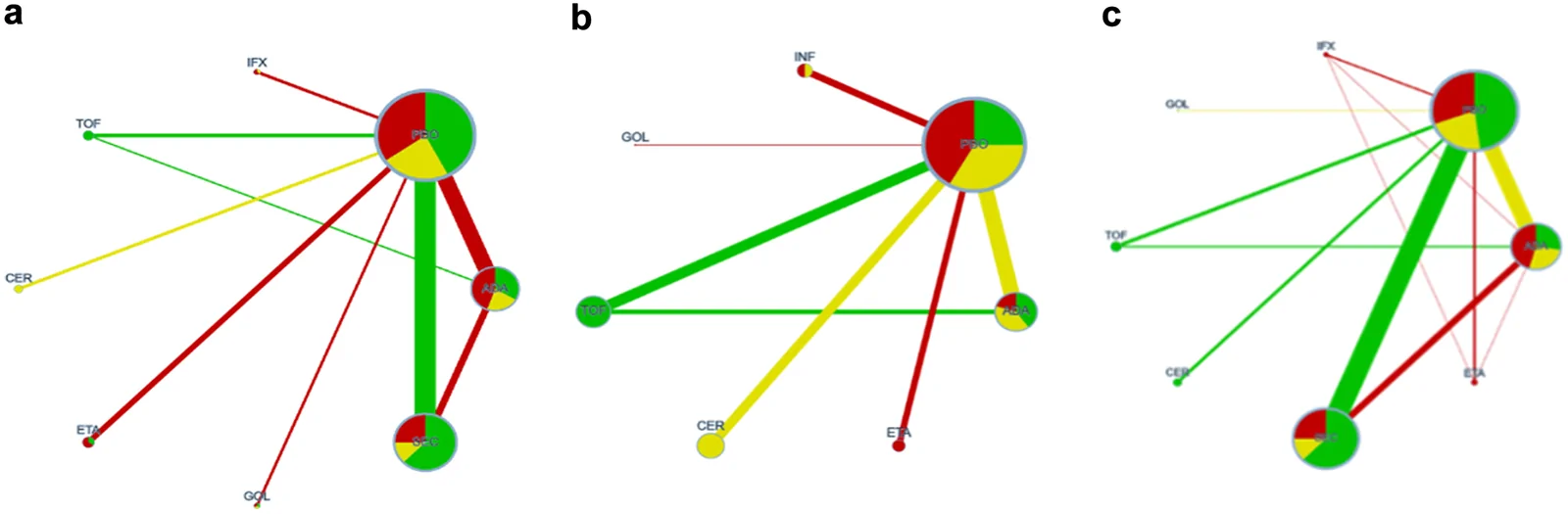
Network diagram of the primary outcomes evaluated at 6-month follow-up in included randomized clinical trials. The size of nodes and lines is proportional to the number of participants allocated to each intervention and comparison. The colors of the nodes and lines correspond to the risk of bias of the included studies (green—Low risk; yellow—some concerns; and red—high risk). **a** - ACR 50; **b** - PsARC; **c** - Serious adverse events. *Abbreviations*: *ADA* adalimumab; *CER* certolizumab pegol; *ETA* etanercept; *GOL* golimumab; *IFN* infliximab; *SEC* secukinumab; *PBO* placebo; *TOF* tofacitinib

Figure 2 displays the network diagrams for the primary outcomes (ACR50, PsARC and serious adverse events).

### Characteristics of the included studies

We included 33 RCTs, comprising 11,034 participants, with a median sample size of 252 participants per study. Among the included RCTs, four were not multicenter studies, and two were not funded by the pharmaceutical industry. The median age of participants was 48 years, and 50% of them were women. The median duration of symptoms was 6 years, and 15 RCTs included only participants who had previously undergone treatment with biologics [20–34].

In terms of comparisons, 30 RCTs compared biologics (with different approved doses pooled into a single treatment arm) with placebo (either MTX or no treatment): Adalimumab (n = 8); Etanercept (n = 3); Infliximab (n = 3); Golimumab (n = 4); Secukinumab (n = 10); Certolizumab pegol (n = 1); Tofacitinib (n = 1)

One RCT compared different drugs with each other (Adalimumab versus Secukinumab, while one had more than two treatment arms comparing drugs with each other biologics and placebo (Adalimumab versus Tofacitinib versus placebo), and one had more than two treatment arms, comparing drugs with each other (Adalimumab versus Etanercept versus Infliximab).

Among the primary outcomes assessed, 28 of the 33 RCTs evaluated the ACR 50 outcome, 13 assessed the PsARC outcome and 25 assessed serious adverse events. More detailed information on the characteristics of the RCTs is provided in Table 1 and Additional file Tables S3, S4 and S5.

**Table 1:** Summary of randomized clinical trials’ characteristics included in the systematic review

**General information**		
	N. of RCTs included	33
	N. total	11,034
**Characteristics of studies**		
	Median of sample size	252
	Minimum sample size	22
	Maximum sample size	1705
	N. of studies with sample size per arm N ≥ 100	19
	N. multicenter studies	29
	N. studies that analysed outcomes at 4 weeks follow-up	2
	N. studies that analysed outcomes at ≤12 weeks follow-up	6
	N. studies that analysed outcomes at ≤16 weeks follow-up	8
	N. studies that analysed outcomes between 22 and 24 weeks of follow-up	15
	N. studies that analysed outcomes between 48 and 52 weeks of follow-up	2
	N. studies funding by pharmaceutical companies	31
**Characteristics of participants**		
	Age (mean (SD))	47.70 (2.07)
	Symptoms duration in years (median (IQR))	6.34 (2.64)
	Weight in Kg (median (IQR))	85.70 (1.52)
	% of women (median (IQR))	50.00 (10.40)
	N. of studies that included participants with previous biological treatment	15
	% participants with axial or enthesitis	64.00 (15.34)
	% participants with peripheral arthritis (including dactylitis)	37.14 (19.11)
**Intervention arms**		
	N. of studies with Adalimumab	11
	N. of studies with Certolizumab Pegol	1
	N. of studies with Etanercept	4
	N. of studies with Golimumab	4
	N. of studies with Infliximab	4
	N. of studies with Secukinumab	11
	N. of studies with Tofacitinib	2
	N. of studies with MTX isolated	1
	N. studies with no treatment	2
	N. studies with placebo	28
**Outcomes analysed**		
	N. of studies that analysed ACR 50	28
	N. of studies that analysed PsARC	13
	N. of studies that analysed HAQ-DI continuous	29
	N. of studies that analysed HAQ-DI dichotomous	8
	N. of studies that analysed disease activity (DAPSA, DAS28, CDAI or MDA) continuous	20
	N. of studies that analysed disease activity (DAPSA, DAS28, CDAI or MDA) dichotomous	14
	N. of studies that analysed pain	14
	N. of studies that analysed quality of life	22
	N. of studies that analysed skin disease (PASI) continuous	10
	N. of studies that analysed skin disease (PASI) dichotomous	23
	N. of studies that analysed serious adverse events	25
	N. of studies that analysed total adverse events	22

*Abbreviations*: *N* number, *IQR* interquartile range

### Risk of bias in studies

**Fig. 3:**
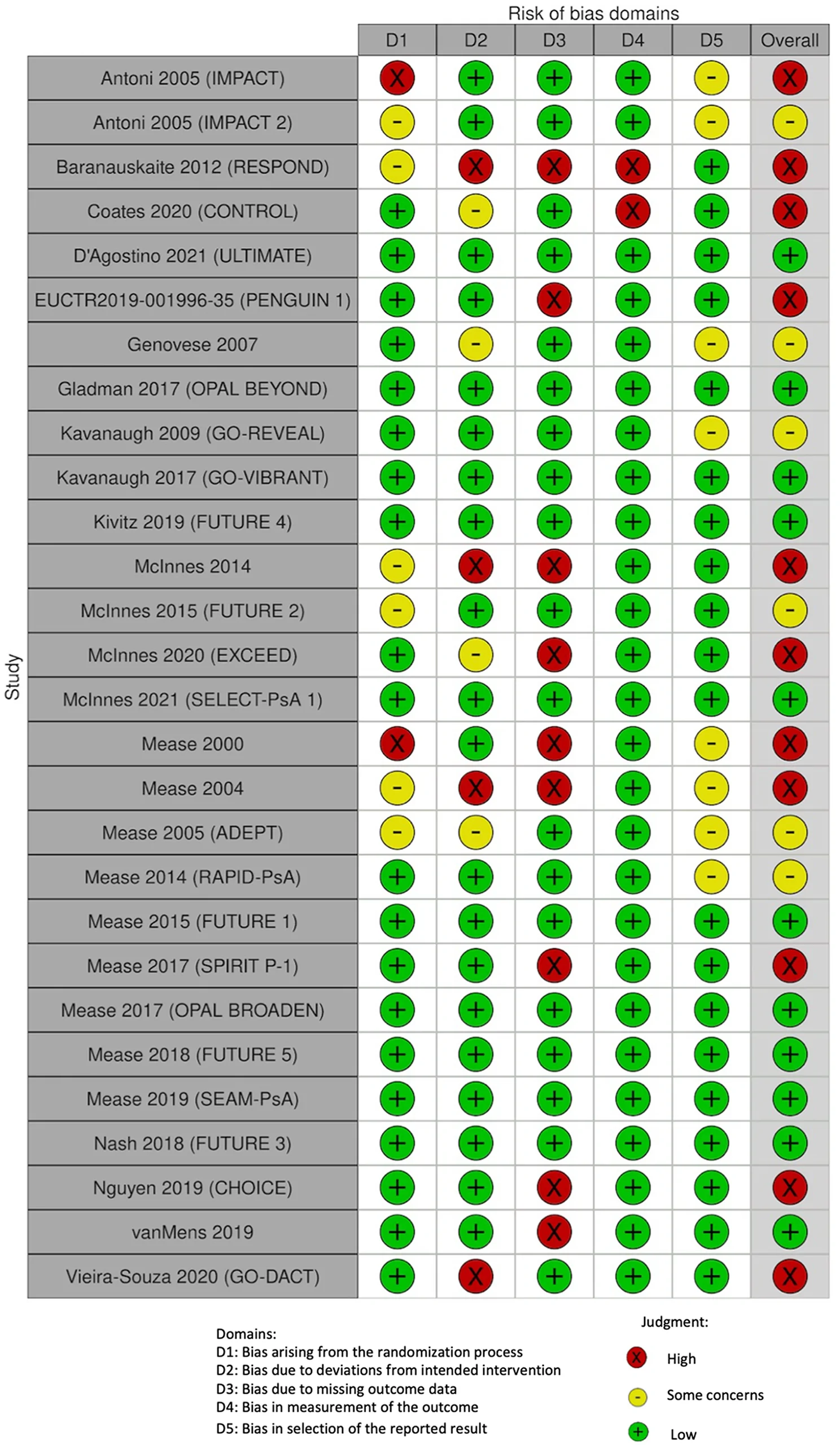

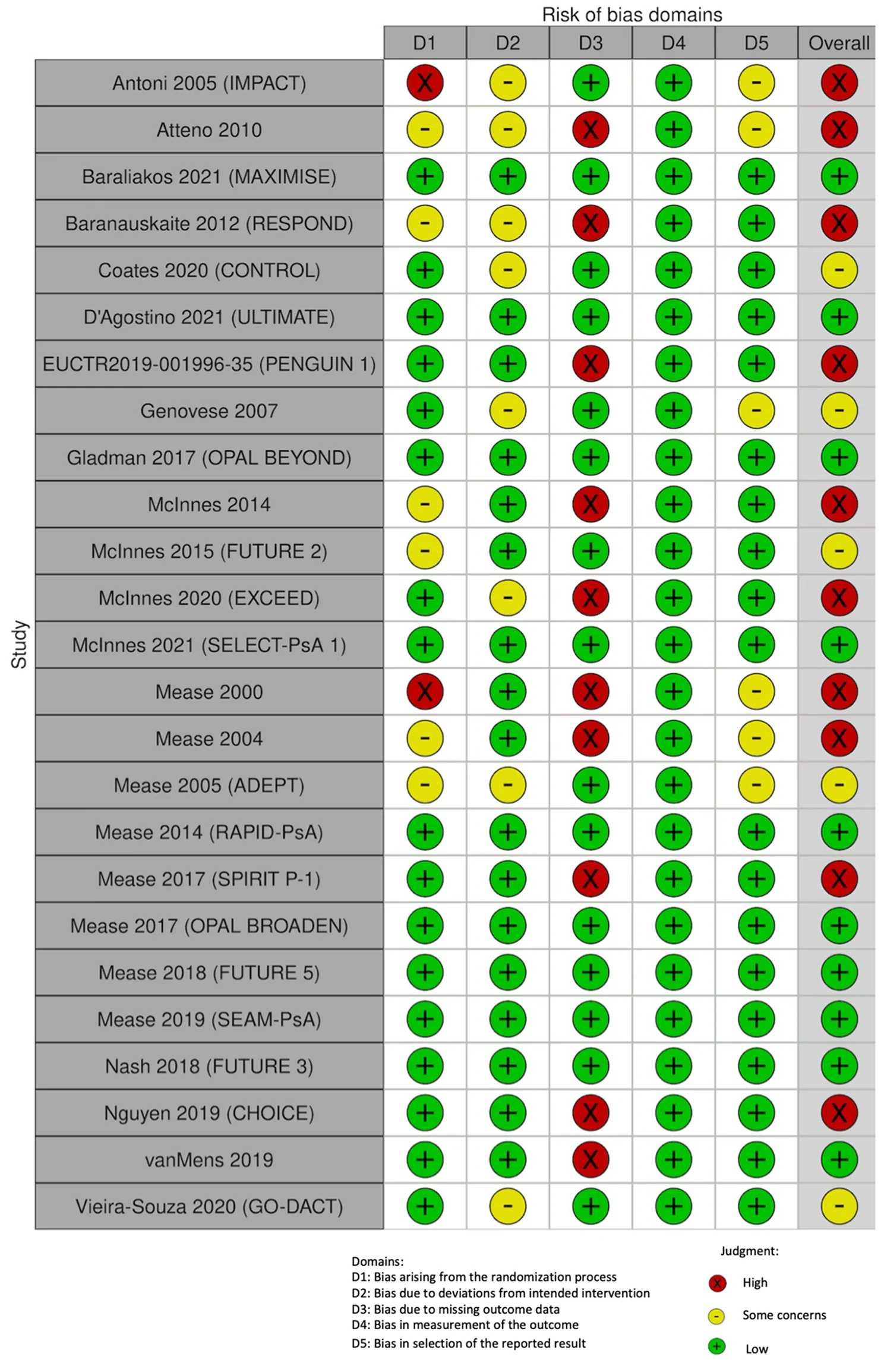
Risk of bias assessment (RoB 2) at outcome level of the randomized clinical trials included. **a** - ACR 50 and PsARC; **b** - Serious adverse events

We used the Cochrane Risk of Bias Tool 2.0 (RoB 2) to assess the risk of bias of RCTs at the level of the primary outcomes. Out of the 28 studies that evaluated the primary efficacy outcomes (ACR 50 and/or PsARC), 11 were classified as having a low risk of bias [24, 28, 29, 31, 32, 34–39], 6 as some concerns [23, 26, 33, 40–42] and 11 as a high risk of bias [20, 25, 27, 30, 43–49]. The domain with most limitation was the bias due to missing data, with 9 out of the 28 (32%) RCTs judged as high risk of bias. Figure 3a presents the summary of the risk of bias assessment at the outcome level.

Regarding the primary safety outcome (serious adverse events), out of 25 RCTs evaluated, 10 were classified as low risk of bias [28, 29, 31, 32, 35, 36, 38, 39, 42, 46, 50], 5 as some concerns [23, 26, 41, 44, 48] and 10 as high risk of bias [20, 21, 25, 27, 30, 43, 45–47, 49]. The domain with most limitation was bias due to missing data, with 10 out of the 25 (40%) included RCTs judged as high risk of bias. Detailed information on the assessment of the risk of bias in the RCTs for the primary safety outcome can be found in Fig. 3b.

### Results of primary outcomes

#### Disease response (ACR 50)

The NMA included 26 RCTs [20, 23, 24, 26–36, 38–49] that assessed the ACR 50 outcome at 6-month follow-up. The ‘Summary of Findings’ Table 2 summarizes the results for the outcome ACR 50.

**Table 2:** Summary of findings 1: biological treatments compared to placebo for Psoriatic Arthritis—ACR50

Summary of findings						
Biological treatment effects to ACR 50 outcome (6-month follow-up) in the treatment of Psoriatic Arthritis patients						
**Population**: Psoriatic Arthritis patients **Intervention**: Biological treatments (Adalimumab, Etanercept, Infliximab, Golimumab, Secukinumab, Certolizumab pegol) and synthetic target-specific drugs (Tofacitinib) **Comparator (reference)**: Placebo						
Biological treatments	Anticipated absolute effect^*^ (95% CI)		Relative effect (95% CI)	No of participants (studies)	Certainty of evidence (GRADE)	Ranking
	Without intervention	With intervention				
Secukinumab	8 per 100	32 per 100 (22–46)	RR 4.13 (2.87–5.94)	2535 (7 RCTs)	⨁⨁⨁⨁ High	1º - P-score = 0.77
Infliximab	12 per 100	44 per 100 (22–88)	RR 3.79 (1.87–7.68)	403 (3 RCTs)	⨁◯◯◯ Very low	2º - P-score = 0.66
Adalimumab	13 per 100	46 per 100 (32–67)	RR 3.58 (2.47–5.19)	1957 (7 RCTs)	⨁⨁⨁⨁ High	3º - P-score = 0.62
Certolizumab pegol	13 per 100	42 per 100 (17–100)	RR 3.37 (1.34–8.49)	409 (1 RCT)	⨁⨁◯◯ Low	4º - P-score = 0.57
Golimumab	12 per 100	38 per 100 (20–71)	RR 3.15 (1.68–5.90)	347 (3 RCTs)	⨁⨁⨁⨁ High	5º - P-score = 0.51
Tofacitinib	12 per 100	38 per 100 (21–71)	RR 3.11 (1.67–5.77)	472 (2 RCTs)	⨁⨁⨁◯ Moderate	6º - P-score = 0.49
Etanercept	24 per 100	65 per 100 (34–100)	RR 2.71 (1.42–5.18)	623 (3 RCTs)	⨁⨁⨁◯ Moderate	7º - P-score = 0.39
Placebo	–	–	Reference comparator	–	–	–

*The risk of intervention group (and 95% confidence interval) is based on the assumed risk of the comparator group and the **relative effect** of the intervention (and 95% confidence interval)

*CI* confidence interval, *RR* risk ratio

**GRADE working group grades of evidence**

**High certainty:** we are very confident that the true effect lies close to that of the estimate of the effect

**Moderate certainty:** we are moderately confident in the effect estimate: the true effect is likely to be close to the estimate of the effect, but there is a possibility that it is substantially different

**Low certainty:** our confidence in the effect estimate is limited: the true effect may be substantially different from the estimate of the effect

**Very low certainty:** we have very little confidence in the effect estimate: the true effect is likely to be substantially different from the estimate of effect

The forest plot presented in Additional file—Fig. S1, showed that all drugs were superior to placebo. The league table (Additional file—Table S6) showed that there was no difference between Adalimumab, Etanercept, Infliximab, Golimumab, Secukinumab, Certolizumab pegol and Tofacitinib to ACR 50 outcome at 6-months follow-up. The certainty of evidence assessment for each comparison was performed using CINeMA and classified as high (green), moderate (blue), low (yellow) and very low (red).

Ranking analysis using the *P-score* (Additional file—Fig. S2) suggests that Secukinumab has better probability to achieve the ACR 50 in comparison with the other drugs at 6-month follow-up (versus placebo: RR 4.13; 95% CI 2.87–5.94; *P-score* = 0.77; high certainty of evidence), followed by Infliximab (versus placebo: RR 3.79; 95% CI 1.87–7.68; *P-score* = 0.66; very low certainty of evidence), Adalimumab (versus placebo: RR 3.58; 95% CI 2.47 a 5.19; *P-score* = 0.62; high certainty of evidence), Certolizumab pegol (versus placebo: RR 3.37; 95% CI 1.34–8.49; *P-score* = 0.57; very low of evidence), Golimumab (versus placebo: RR 3.15; 95% CI 1.68–5.90; *P-score* = 0.51; high certainty of evidence), Tofacitinib (versus placebo: RR 3.11; 95% CI 1.67–5.77; *P-score* = 0.49; moderate certainty of evidence), and Etanercept (versus placebo: RR 2.71; 95% CI 1.42 a 5.18; *P-score* = 0.39; moderate certainty of evidence).

At 12-month follow-up, one study compared directly the Adalimumab and Secukinumab and found no significant difference between the drugs in terms of achieving ACR 50 outcome (RR = 0.92; 95% CI 0.79–1.06) [46].

#### Disease response (PsARC)

The NMA included eleven RCTs [20, 23, 27, 31, 33, 36, 41, 42, 45, 47, 48] that assessed the PsARC outcome at 6-month follow-up. The ‘Summary of Findings’ Table 3 summarizes the results for the outcome PsARC.

**Table 3:** Summary of findings 2: biological treatments compared to placebo for Psoriatic Arthritis—PsARC

Summary of findings						
Biological treatment effects to PsARC outcome (6-month follow-up) in the treatment of Psoriatic Arthritis patients						
**Population**: Psoriatic Arthritis patients **Intervention**: Biological treatments (Adalimumab, Etanercept, Infliximab, Golimumab, Certolizumab pegol) and synthetic target-specific drugs (Tofacitinib) **Comparator (reference)**: Placebo						
Biological treatments	Anticipated absolute effect^*^ (95% CI)		Relative effect (95% CI)	No of participants (studies)	Certainty of evidence (GRADE)	Ranking
	Without intervention	With intervention				
Etanercept	23 per 100	76 per 100 (46–100)	RR 3.27 (1.98–5.42)	265 (2 RCTs)	⨁⨁◯◯ Low	1º - P-score = 0.91
Infliximab	28 per 100	75 per 100 (47–100)	RR 2.64 (1.65–4.22)	304 (2 RCTs)	⨁⨁◯◯ Low	2º - P-score = 0.78
Certolizumab pegol	33 per 100	78 per 100 (44–100)	RR 2.35 (1.33–4.14)	409 (1 RCT)	⨁⨁◯◯ Low	3º - P-score = 0.68
Adalimumab	31 per 100	61 per 100 (44–84)	RR 1.95 (1.41–2.69)	652 (4 RCTs)	⨁⨁⨁◯ Moderate	4º - P-score = 0.53
Tofacitinib	36 per 100	58 per 100 (39–86)	RR 1.62 (1.09–2.40)	474 (2 RCTs)	⨁◯◯◯ Very low	5º - P-score = 0.37
Golimumab	73 per 100	85 per 100 (47–100)	RR 1.17 (0.64–2.13)	42 (1 RCT)	⨁◯◯◯ Very low	6º - P-score = 0.17
Placebo	–	–	Reference comparator	–	–	–

*The risk of intervention group (and 95% confidence interval) is based on the assumed risk of the comparator group and the **relative effect** of the intervention (and 95% confidence interval)

*CI* confidence interval, *RR* risk ratio

**GRADE working group grades of evidence**

**High certainty:** we are very confident that the true effect lies close to that of the estimate of the effect

**Moderate certainty:** we are moderately confident in the effect estimate: the true effect is likely to be close to the estimate of the effect, but there is a possibility that it is substantially different

**Low certainty:** our confidence in the effect estimate is limited: the true effect may be substantially different from the estimate of the effect

**Very low certainty:** we have very little confidence in the effect estimate: the true effect is likely to be substantially different from the estimate of effect

The forest plot indicated that all drugs were superior to placebo, except for Golimumab (Additional file—Fig. S3). The league table in Additional file—Table S7 for the NMA analysis of PsARC outcome at the 6-month follow-up showed no significant difference between Adalimumab, Etanercept, Infliximab, Certolizumab pegol and Tofacitinib. However, Etanercept and Infliximab were superior to Golimumab (RR 2.80; 95% CI 1.28–6.14 and RR 1.66; 95% CI 1.05–1.79, respectively). The certainty of evidence assessment for each comparison was performed using CINeMA and classified as moderate (blue), low (yellow) e very low (red).

The ranking analysis (*P-score*) presented in Additional file—Fig. S4 shows that Etanercept had the highest probability of achieving PsARC compared to the other drugs at 6-month follow-up (versus placebo: RR 3.27; 95% CI 1.98–5.42; *P-score* = 0.91; very low certainty of evidence). This was followed by Infliximab (versus placebo: RR 2.64; 95% CI 1.65–4.22; *P-score* = 0.78; very low certainty of evidence), Certolizumab pegol (versus placebo: RR 2.35; 95% CI 1.33–4.14; *P-score* = 0.68; low certainty of evidence), Adalimumab (versus placebo: RR 1.95; 95% CI 1.41–2.69; *P-score* = 0.53; moderate certainty of evidence), Tofacitinib (versus placebo: RR 1.62; 95% CI 1.09–2.40; *P-score* = 0.37; very low certainty of evidence), and Golimumab (versus placebo: RR 1.17; 95% CI 0.64 a 2.13; *P-score* = 0.17; very low certainty of evidence).

#### Serious adverse events

Twenty four RCTs [20, 21, 23, 26–32, 34–36, 38, 41–50] were included in the NMA that evaluated serious adverse events. The ‘Summary of Findings’ Table 4 summarize the results for the outcome serious adverse events.

**Table 4:** Summary of findings 2: biological treatments compared to placebo for Psoriatic Arthritis—serious adverse events

Summary of findings						
Biological treatment effects to serious adverse events outcome in the treatment of Psoriatic Arthritis patients						
**Population**: Psoriatic Arthritis patients **Intervention**: Biological treatments (Adalimumab, Etanercept, Infliximab, Golimumab, Secukinumab, Certolizumab pegol) and synthetic target-specific drugs (Tofacitinib) **Comparator (reference)**: Placebo						
Biological treatments	Anticipated absolute effect^*^ (95% CI)		Relative Effect (95% CI)	№ of participants (studies)	Certainty of evidence (GRADE)	Ranking
	Without intervention	With intervention				
Golimumab	4 per 100	1 per 100 (0–13)	RR 0.34 (0.04–3.17)	93 (2 RCTs)	⨁◯◯◯ Very low	1º - P-score = 0.83
Secukinumab	4 per 100	3 per 100 (2–5)	RR 0.85 (0.59–1.23)	2706 (7 RCTs)	⨁⨁◯◯ Low	2º - P-score = 0.64
Adalimumab	2 per 100	2 per 100 (1–3)	RR 0.89 (0.59–1.34)	1957 (6 RCTs)	⨁◯◯◯ Very low	3º - P-score = 0.60
Etanercept	4 per 100	3 per 100 (1–11)	RR 0.86 (0.25–3.01)	265 (2 RCTs)	⨁◯◯◯ Very low	4º - P-score = 0.58
Tofacitinib	2 per 100	2 per 100 (1–8)	RR 1.23 (0.30–5.00)	474 (2 RCTs)	⨁◯◯◯ Very low	5º - P-score = 0.41
Infliximab	1 per 100	2 per 100 (0–15)	RR 2.00 (0.26–15.24)	214 (2 RCTs)	⨁◯◯◯ Very low	6º - P-score = 0.26
Certolizumab pegol	4 per 100	8 per 100 (3–19)	RR 1.74 (0.72–4.22)	409 (1 RCT)	⨁⨁◯◯ Low	7º - P-score = 0.20
Placebo	–	–	Reference comparator	–	–	–

*The risk of intervention group (and 95% confidence interval) is based on the assumed risk of the comparator group and the **relative effect** of the intervention (and 95% confidence interval)

*CI* confidence interval, *RR* risk ratio

**GRADE working group grades of evidence**

**High certainty:** we are very confident that the true effect lies close to that of the estimate of the effect

**Moderate certainty:** we are moderately confident in the effect estimate: the true effect is likely to be close to the estimate of the effect, but there is a possibility that it is substantially different

**Low certainty:** our confidence in the effect estimate is limited: the true effect may be substantially different from the estimate of the effect

**Very low certainty:** we have very little confidence in the effect estimate: the true effect is likely to be substantially different from the estimate of effect

The forest plot (Additional file—Fig. S5) indicated no significant differences between the drugs and placebo. The league table in Additional file—Table S8 demonstrated there were no significant differences between Adalimumab, Etanercept, Infliximab, Golimumab, Secukinumab, Certolizumab pegol and Tofacitinib in terms of serious adverse events. The certainty of evidence analysis for each comparison was performed by CINeMA and classified as low (yellow) and very low (red).

According to the ranking of the evaluated interventions (Additional file—Fig. S6), Golimumab had the highest *P-score* in terms of occurrence of serious adverse events (versus placebo: RR 0.34; 95% CI 0.04–3.17; *P-score* = 0.83; very low certainty of evidence), followed by Secukinumab (versus placebo: RR 0.85; 95% CI 0.59–1.23; *P-score* = 0.64; low certainty of evidence), Adalimumab (versus placebo: RR 0.89; 95% CI 0.59–1.34; *P-score* = 0.60; very low certainty of evidence) Etanercept (versus placebo: RR 0.86; 95% CI 0.25–3.01; *P-score* = 0.58; very low of certainty of evidence), Placebo, Tofacitinib (versus placebo: RR 1.23; 95% CI 0.30–5.00; *P-score* = 0.41; very low certainty of evidence), Infliximab (versus placebo: RR 2.00; 95% CI 0.26–15.24; *P-score* = 0.26; very low certainty of evidence) and Certolizumab pegol (versus placebo: RR 1.74; 95% CI 0.26–15.24; *P-score* = 0.20; low certainty of evidence).

### Results of secondary outcomes

Additional file—Fig. S7 presents the network diagrams for the secondary outcomes.

#### Function

The NMA that evaluated the outcome function (measured by HAQ-DI questionnaire) included six RCTs for dichotomous data [24, 30, 31, 33, 36, 44] and two for continuous data [21, 46]. In the analysis of dichotomous data, all drugs were superior to placebo (Additional file—Fig. S8). There was no significant difference between the drugs, except for Infliximab, which was superior Adalimumab (Additional file—Table S9a). The ranking of interventions (*P-score*) demonstrated that Infliximab had the highest probability of improving function, followed by Tofacitinib, Adalimumab, and Secukinumab (Additional file—Fig. S9a). In the continuous data analysis, no significant difference was found between Adalimumab, Etanercept, Infliximab and Secukinumab (Additional file–Table S9b). The ranking of interventions (*P-score*) demonstrated that Etanercept, Infliximab and Secukinumab had the highest probability of improving function (Additional file—Fig. S9b).

At 12-month follow-up, two RCTs [21, 46] analyzed the function outcome using the HAQ-DI questionnaire and found no significant difference between Adalimumab, Etanercept, Infliximab and Secukinumab (Additional file—Table S10).

#### Disease activity

The NMA evaluating disease activity (measured by DAPSA or DAS28 or MDA) at 6-month follow-up included 14 RCTs [20, 24, 29, 31, 32, 36, 38, 39, 42–45, 48, 49] for dichotomous data and 18 RCTs [20, 24, 26, 28–31, 34, 36, 38–40, 43–45, 48, 49, 51] for continuous data. All drugs were superior to placebo except for Etanercept (Additional file—Fig. S10).

In dichotomous data analysis, Certolizumab pegol and Secukinumab were superior to Etanercept and Golimumab (Additional file—Table S11a). In terms of ranking (*P-score*), Certolizumab pegol had the highest probability of improving disease activity, followed by Secukinumab, Infliximab, Adalimumab, Tofacitinib and Etanercept (Additional file—Fig. S11a).

In the analysis of continuous data (DAS28), Infliximab was superior to the others interventions (Additional file—Table S11b). Based on the ranking (*P-score*), Infliximab had the highest probability of improving disease activity, followed by Adalimumab, Golimumab, Tofacitinib, Secukinumab and Etanercept (Additional file—S11b).

At 12-month follow-up, one study [46] evaluated the disease activity (DAS28) and showed that Secukinumab was superior to Adalimumab (RR = 1.12; 95% CI 1.03–1.20).

#### Pain

The NMA evaluating pain at 6-month follow-up included 14 RCT [20, 23, 28, 30–34, 36, 38, 42, 43, 50, 52]. Among the interventions evaluated (Adalimumab, Infliximab, Golimumab, Secukinumab, Certolizumab pegol and Tofacitinib), only Infliximab was superior to placebo (Additional file—Fig. S12). No significant differences were found between the drugs (Additional file—Table S12). The ranking analysis (*P-score*) indicated that Golimumab and Infliximab had the highest probability of improving pain, followed by Certolizumab pegol, Tofacitinib, Adalimumab and Secukinumab (Additional file—Fig. S13).

#### Quality of life

The SF-36 PCS was used to measure the quality of life outcome in 20 RCTs at6-months follow-up [23, 24, 26, 28, 30–34, 36, 38–42, 44, 45, 47, 49, 52]. The NMA demonstrated that all evaluated drugs (Adalimumab, Etanercept, Infliximab, Golimumab, Secukinumab, Certolizumab pegol and Tofacitinib) were superior to placebo (Additional file—Fig. S14). No significant differences were found in the comparisons between the drugs (Additional file—Table S13). Regarding the ranking analysis (*P-score*), Infliximab had the highest probability of improving quality of life, followed by Certolizumab pegol, Etanercept, Adalimumab, Golimumab, Secukinumab and, Tofacitinib (Additional file—Fig. S15).

#### Skin disease

The outcome skin disease at a 6 month follow-up was analyzed in 21 RCTs [20, 24, 26–34, 36, 40–45, 47–49] using dichotomous data (PASI 75) and in 6 RCT [20, 22, 38, 45, 48, 51] using continuous data analysis (PASI score). The NMA results for PASI 75 (dichotomous) showed that all drugs analyzed (Adalimumab, Etanercept, Infliximab, Golimumab, Secukinumab, Certolizumab pegol e Tofacitinib) were superior to placebo (Additional file—Fig. S16a). When compared to each other, there were no significant differences (Additional file—Table S14a). The ranking analysis (*P-score*) indicated that Etanercept had the highest probability of improving skin disease, followed by Secukinumab, Certolizumab pegol, Infliximab, Adalimumab, Golimumab e Tofacitinib (Additional file—Fig. S17a).

In the NMA for PASI score (continuous), Infliximab was superior to placebo, while Adalimumab and Golimumab showed no significant differences compared to placebo (Additional file—Fig. S16b). When compared to each other, Infliximab was superior to Adalimumab and Golimumab, while there was no significant difference in the comparison between Adalimumab and Golimumab (Additional file—Table S14b). The ranking (*P-score*) indicated that Infliximab had the highest probability of improving skin activity disease, followed by Adalimumab and Golimumab (Additional file—Fig. S17b).

One study compared the effectiveness of Secukinumab and Adalimumab in treating skin disease using dichotomous data (PASI 75) at 12-month follow-up [46]. The results indicated that Secukinumab was superior to Adalimumab. Another study measured skin disease using continuous data (PASI score) at 12-month follow-up, comparing Adalimumab, Etanercept and Infliximab [21]. The results demonstrated that Etanercept was more effective than Adalimumab e Infliximab (Additional file—Table S15).

#### Total adverse events

A total of 21 RCTs [20, 21, 23, 26, 28–32, 34–36, 38, 42–46, 48–50] were included in the NMA of total adverse events. When comparing the drugs to each other, no significant differences were observed (Additional file—Table S16). The analysis showed no significant differences between drugs (Adalimumab, Etanercept, Golimumab, Secukinumab, Certolizumab pegol and Tofacitinib) and the placebo with respect to the risk of total adverse events, expect for Infliximab, which had a higher risk compared with placebo (Additional file—Fig. S18). The ranking analysis (*P-score*), indicated that Secukinumab had the highest probability of being the safest option in terms of the occurrence of total adverse events, followed by Certolizumab pegol, Golimumab, Adalimumab, Etanercept, Tofacitinib, and Infliximab (Additional file—Fig. S19).

### Publication bias

**Fig. 4:**
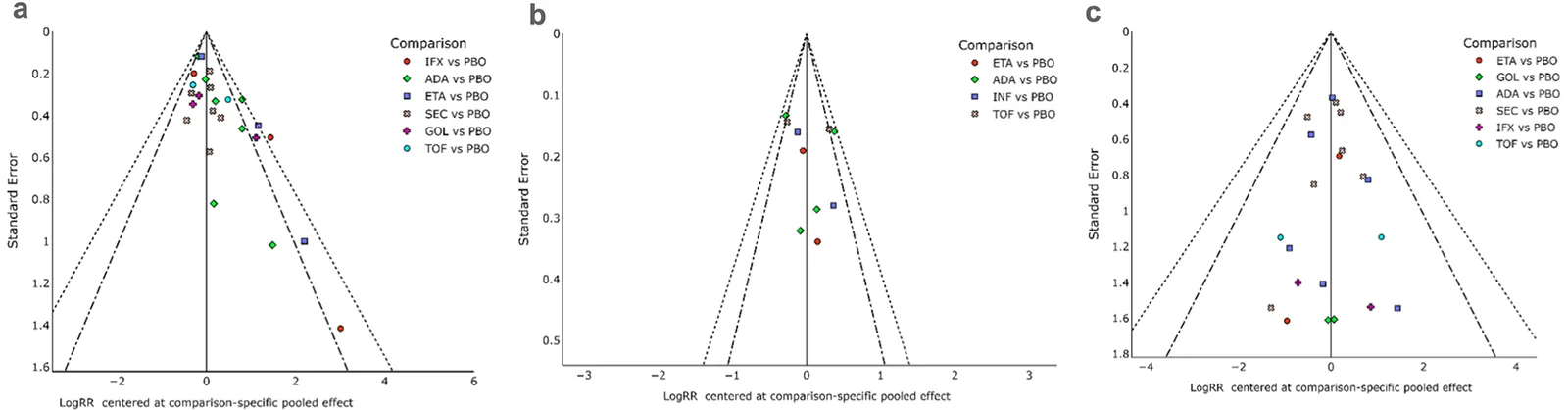
Funnel plot of primary outcomes analyzed in network meta-analysis at 6-month follow-up. **a** - ACR 50; **b** - PsARC; **c** - Serious adverse events. *Abbreviations:* *ADA* adalimumab, *ETA* etanercept, *GOL* golimumab, *IFN* infliximab, *SEC* secukinumab, *PBO* placebo, *TOF* tofacitinib

We assessed the publication bias of primary outcomes of the RCTs using the funnel plot, when possible (at least 10 studies for each outcome). These graphs were created by analyzing the outcomes at 6-month follow-up and using the placebo intervention as a reference. An asymmetry in the funnel plot was observed for the ACR 50 outcome (Fig. 4a), indicating a possible publication bias, which could lead to an overestimation of the estimated effect of the NMA. However, for the PsARC (Fig. 4b) and serious adverse events (Fig. 4c) outcomes, no important asymmetry was observed, suggesting that there is probably no publication bias.

### Additional analysis

Sensitivity analyses were performed for primary outcomes ACR50 and serious adverse events, including only RCTs with low risk of bias and sample size of at least 100 participants per treatment arm. However, due to insufficient data, sensitivity analyses investigating industry funding and age of participants could not be carried out. In turn, although five multicenter trials included centers in Brazil, it was still not feasible to perform analyses that exclusively included Brazilians [32, 34, 36, 42, 44].

The NMA for the outcome ACR50 at 6-month follow-up, which included RCTs with low risk of bias, evaluated the drugs Adalimumab, Etanercept, Golimumab, Secukinumab, and Tofacitinib. Similar to the main analysis, all drugs were found to be superior to placebo, with Secukinumab having the highest probability of achieving ACR50 compared to other drugs. However, Tofacitinib moved up in the ranking from penultimate to second place.

The sensitivity analysis including RCTs with sample size of at least 100 participants per treatment arm evaluated the same drugs as the main analysis. Again, all drugs were superior to placebo, but some differences were found in the ranking. In contrast to the main analysis, Infliximab appeared to have a better probability of achieving ACR50 in the sensitivity analysis, whereas Secukinumab was in the first position in the main analysis.

The sensitivity analyses for serious adverse outcomes yielded similar results to the main analysis.

### Certainty in the evidence

The assessment of the certainty of evidence for the outcome ACR50 indicated that the confidence level varied from very low to high (Additional file—Table S17), depending on the comparison analyzed. For the outcome PsARC, the confidence level was classified as very low to moderate, depending on the comparison analyzed (Additional file—Table S18), while for serious adverse events range from very low to low (Additional file—Table S19).

Certain domains had a greater impact on the downgrading of the evidence. In some comparisons, the most common reasons for downgrading two levels were within-study bias and imprecision. We also downgraded in one level the certainty of evidence in some comparison due to indirectness and heterogeneity. We detected no reporting bias in all assessments, and incoherence was present in only one comparison (adalimumab versus placebo; serious adverse event).

## Discussion

This systematic review conducted a network meta-analysis (33 RCTs, n = 11,034) to compare the efficacy and safety of all biological and specific target synthetic drugs used in the treatment of psoriatic arthritis under the Brazilian Unified Health System. All drugs outperformed placebo in terms of disease response outcome (ACR 50—6-month follow-up), with Secukinumab, Infliximab and Adalimumab presenting the highest *P-score* values (ranking). In the assessment of the other primary efficacy outcome (PsARC) at the same follow-up, all evaluated drugs, except for Golimumab were superior to placebo, with Etanercept, Infliximab and Certolizumab pegol presenting the highest *P-score* values.

The risk of serious adverse events was not significant different between the evaluated drugs and placebo, which is relevant considering that patients’ main concerns when using these drugs are related to side effects and long-term safety [53]. The *P-score* analysis revealed that Golimumab, Secukinumab, and Adalimumab had the highest values for this outcome, while Infliximab and Certolizumab pegol had the lowest. These findings are consistent with a previous observational study conducted in Brazil, which indicated that patients taking Infliximab had lower medication persistence rates than those taking Adalimumab [54].

The analysis of secondary outcomes, including HAQ-DI, disease activity measured by DAPSA, DAS28 or MDA, quality of life, and skin disease, demonstrated the evaluated drugs were superior to placebo. However, there were no significant differences when comparing the drugs to each other, except for the disease activity outcome, where Secukinumab and Certolizumab pegol outperformed Etanercept and Golimumab. Infliximab was the only one that was superior to placebo in improving pain. Regarding the risk of total adverse events, there was no significant difference between the evaluated drugs and placebo, except for Infliximab, which presented a higher risk compared to placebo.

This systematic review’s findings were compared with four other systematic reviews that used NMA of RCTs to evaluate treatments for patients diagnosed with psoriatic arthritis [8, 9, 11, 55]. It is important to note that these previous reviews included different drugs compared to our review. Our review found that all drugs were more effective than placebo for the disease response outcome (ACR 50), which is consistent with the reviews conducted by Ruyssen-Witrand et al. [11], McInnes et al. [8, 9]. Ruyssen-Witrand et al.’s systematic review found that Infliximab, Golimumab and Etanercept were the most effective interventions for ACR outcomes compared to placebo. In contrast, in our systematic review, Secukinumab showed greater efficacy, followed by Infliximab and Adalimumab [11]. Additionally, McInnes et al.’s [9] study found that Infliximab and Etanercept were the most effective interventions for ACR outcome. The differences in the results found may be attributed to Ruyssen-Witrand et al.’s [11] and McInnes et al.’s [9] reviews combining the effects of ACR 20, ACR 50, and ACR 70, while our review only evaluated the effect of ACR 50.

Our systematic review found that all drugs, except for Golimumab, were more effective than placebo for the disease response outcome (PsARC), with Certolizumab pegol, Etanercept and Infliximab being the most effective drugs. Similarly, Ruyssen-Witrand et al. [11] also reported that Etanercept and Infliximab were among the most effective interventions, whereas McInnes et al. [8] also observed that all treatments demonstrated superiority over placebo. The reviews by Lu et al. [10] and McInnes et al. [9] did not present the results regarding PsARC.

Concerning results of skin disease, our findings were quite similar to those of other systematic reviews [9, 11]. Ruyssen-Witrand et al. [11] also observed that Infliximab had significant benefits for PASI score (continuous) compared to other drugs. Similarly, in McInnes et al. [9], among the drugs of interest in our review, Secukinumab (300 mg) and Infliximab showed the most substantial treatment effects compared to placebo for PASI score and PASI 75. In our review, we also found that Infliximab had a greater effect on PASI score, while Secukinumab ranked second among the other drugs for PASI 75.

Regarding the risk of serious adverse events, the result of the systematic review by Lu et al. was similar to our review. No drug showed a significant difference compared to placebo in terms of the risk of serious adverse events, and interventions with the best ranking were also similar [10]. Similarly, the study by Ruyssen-Witrand et al. observed that no treatment had a statistically significant greater or lesser chance of presenting serious adverse events compared to placebo [11]. The reviews by McInnes et al. [8, 9] did not present results regarding serious adverse events.

One of the strengths of this review, in addition to the sensitive and extensive search in the literature, was the rigorous process that we followed throughout the review, in line with the recommendations of the Cochrane Collaboration. For instance, the selection of studies, extracting data, assessing of the risk of bias, and certainty of evidence were all carried out by two independent evaluators. This approach gives us confidence that the results of this review are accurate.

An important limitation of the present systematic review is the potential for publication bias. Specifically, for the ACR 50 outcome, an asymmetry in the funnel plot was observed, suggesting that there may be an overestimation of the effect estimate of the NMA. Additionally, it is worth noting that only 2 of the 33 RCTs included did not receive any type of commercial financing. To address the possibility of this type of bias, we conducted an extensive search for unpublished studies. For the other outcomes, either no asymmetry was observed in the funnel plot evaluation, or it was not possible to evaluate due to the limited number of studies.

Another possible limitation of this review is our approach to studies evaluating different doses of the drugs of interest. We chose only the doses recommended in the Brazilian [6], and in cases where multiple doses of the same drug were approved, they were grouped into a single treatment arm. We also decided not to analyze naïve-patients separately from those who had prior treatment experience with specific target biological and synthetic drugs. We made this decision to avoid limiting the number of studies included in each analysis and potentially reducing the statistical precision in our results. In addition, in some studies we did not have this information available.

Another important aspect to consider is the level of certainty of the evidence presented in our findings. Although moderate to high level evidence was found for the primary outcome ACR 50 in some comparisons, for most PsARC and serious adverse events comparisons, the certainty of evidence ranged from low to very low. This indicates that confidence in the results of these outcomes is limited and should be interpreted with caution. Moreover, greater care is needed in interpreting the results of safety outcomes as there was no long-term follow-up in the RCTs.

Given the methodological limitations identified in our assessment of included RCTs, more well-designed studies with long-term follow-up will contribute to a better understanding of the effects of the evaluated drugs in the treatment of patients with psoriatic arthritis. Furthermore, given that no RCTs were conducted exclusively with Brazilians, it is crucial to make efforts to assess the efficacy and safety of these drugs in Brazil, particularly through pragmatic randomized clinical trials within the context of SUS.

## Conclusion

Secukinumab and Adalimumab may be the preferred drugs options for the treatment for Psoriatic Arthritis, based on the balance between efficacy (high certainty of evidence for ACR 50) and safety (low to very low certainty for serious adverse events). However, the safety findings should be viewed with caution, as they were based on evidence with a low to very low level of certainty, and long-term data were not found. This implies that the balance between benefit and harm may change as new evidence on safety emerges.

## Electronic supplementary material

Below is the link to the electronic supplementary material.

Supplementary Material 1

## Data Availability

All data generated or analysed during this study are included in this published article [and its supplementary information files].

## List of abbreviations

ADA: Adalimumab
bDMARDs: Biological disease-modifying antirheumatic drugs
csDMARD-IR: Disease-modifying antirheumatic drugs
CER: Certolizumab pegol
CI: Confidence interval
DAPSA: Disease activity in Psoriatic Arthritis score
ETA: Etanercept
GOL: Golimumab
HAQ-DI: Health assessment questionnaire disability index
IFN: Infliximab
MD: Mean difference
MDA: Minimal disease activity
MMCDsae: Specific target synthetic drugs
MTX: Methotrexate
NMA: Network meta-analysis
PsA: Psoriatic Arthritis
PBO: Placebo
PROSPERO: International prospective register of systematic reviews
PsARC: Psoriatic Arthritis response criteria
PSORIQOL: Psoriatic Arthritis quality of life
RCT: Randomized clinical trials
RoB 2: Risk of bias da Cochrane 2.0
ROBINS-I: Risk of bias in non-randomised studies – of interventions
RR: Risk ratio
SMD: Standardized mean difference
SUS: Sistema Único de Saúde (Brazilian Unified Health System)
TOF: Tofacitinib
