# Supplementary material for: Effectiveness and safety of biological and target synthetic drugs treatment for psoriatic arthritis: a systematic review with network meta-analysis
Source: Adv Rheumatol. 2024 Mar 21;64:21. doi: 10.1186/s42358-024-00361-3 (PMC13488810; doi:10.1186/s42358-024-00361-3)
Supplement: Supplementary file 1 [file 42358_2024_361_MOESM1_ESM.docx]

**Effectiveness and safety of biological and target synthetic drugs treatment for psoriatic arthritis: a systematic review with network meta-analysis**

**Thais Montezuma^1^, Livia Fernandes Probst^1,2^, Matheus Almeida de Oliveira^1^**

^1^Health Technology Assessment Unit, Hospital Alemão Oswaldo Cruz, Brazil

^2^Professional master’s degree in management and Collective Health. State University of Campinas, Brazil

**Index of Additional file**

This document contains an index of the supplementary material that accompanies our research paper. The supplementary material includes additional tables, figures, and data that were not included in the main paper due to space limitations.

The Additional file is organized as follows:

[Table S1. Search strategies performed in databases	5](#_Toc135747083)

[Table S2. List of excluded records (and the reason for exclusion) in the full-text eligibility analysis	6](#_Toc135747084)

[Table S3. Characteristics of randomized clinical trials included in systematic review	13](#_Toc135747085)

[Table S4. Population characteristics of randomized clinical trials included in systematic review	15](#_Toc135747086)

[Table S5. Interventions characteristics of randomized clinical trials included in systematic review	19](#_Toc135747087)

[Results of Primary outcomes	26](#_Toc135747088)

[Disease response (ACR 50)	26](#_Toc135747089)

[Figure S1. Network meta-analysis estimates of interventions versus placebo for disease response outcome (ACR 50) at 6-month follow-up	26](#_Toc135747090)

[Table S6. League table of relative effects of interventions for disease response outcome (ACR 50) at 6-month follow up	27](#_Toc135747091)

[Figure S2. Ranking (P-score) of interventions for disease response outcome (ACR 50) at 6-month follow-up	28](#_Toc135747092)

[Disease response (PsARC)	29](#_Toc135747093)

[Figure S3. Network meta-analysis estimates of interventions versus placebo for disease response outcome (PsARC) at 6-month follow-up	29](#_Toc135747094)

[Table S7. League table of relative effects of interventions for disease response outcome (PsARC) at 6-month follow up	30](#_Toc135747095)

[Figure S4. Ranking (P-score) of interventions for disease response outcome (PsARC) at 6-month follow-up	31](#_Toc135747096)

[Serious adverse events	32](#_Toc135747097)

[Figure S5. Network meta-analysis estimates of interventions versus placebo for serious adverse events outcome	32](#_Toc135747098)

[Table S8. League table of relative effects of interventions for serious adverse events outcome	33](#_Toc135747099)

[Figure S6. Ranking (P-score) of interventions for serious adverse events outcome	34](#_Toc135747100)

[Results of secondary outcomes	35](#_Toc135747101)

[Figura S7. Network diagram of the secondary outcomes evaluated in included randomized clinical trials	35](#_Toc135747102)

[Function (HAQ-DI)	36](#_Toc135747103)

[Figure S8. Network meta-analysis estimates of interventions versus placebo for function outcome (HAQ-DI)	36](#_Toc135747104)

[Table S9. League table of relative effects of interventions for function outcome (HAQ-DI)	37](#_Toc135747105)

[Figure S9. Ranking of interventions for function outcome (HAQ-DI)	38](#_Toc135747106)

[Table S10. Direct and indirect comparisons between interventions for function outcome measured by Health Assessment Questionnaire Disability Index (HAQ-DI)	39](#_Toc135747107)

[Disease activity	40](#_Toc135747108)

[Figure S10. Network meta-analysis estimates the effect of interventions compared for placebo on disease activity outcome (DAS-28)	40](#_Toc135747109)

[Table S11. League table of relative effects of interventions on disease activity outcome (DAS-28)	41](#_Toc135747110)

[Figure S11. Ranking of interventions based on their effect on disease activity outcome (DAS-28)	42](#_Toc135747111)

[Pain	43](#_Toc135747112)

[Figure S12. Network meta-analysis estimates the effect of interventions compared to placebo on pain outcome	43](#_Toc135747113)

[Table S12. League table of relative effects of interventions on pain outcome (0 to100 scale)	44](#_Toc135747114)

[Figure S13. Ranking of interventions based on their effect on pain outcome	45](#_Toc135747115)

[Quality of life	46](#_Toc135747116)

[Figure S14. Network meta-analysis estimates the effect of interventions compared to placebo on quality of life outcome, as measured by the Form 36 - physical component summary (SF-36 PCS)	46](#_Toc135747117)

[Table S13. League table of relative effects of interventions on quality of life outcome	47](#_Toc135747118)

[Figure S15. Ranking of interventions and placebo based on their effect on quality of life outcome, as measured by Form 36 - physical component summary (SF-36 PCS)	48](#_Toc135747119)

[Skin disease	49](#_Toc135747120)

[Figure S16. Network meta-analysis estimates the effect of interventions compared to placebo on skin disease outcome, as measured by Psoriasis Area and Severity Index (PASI)	49](#_Toc135747121)

[Table S14. League table of relative effects of interventions on skin disease outcome	50](#_Toc135747122)

[Figure S17. Ranking of interventions based on their effect on skin disease outcome, as measured by Psoriasis Area and Severity Index (PASI)	51](#_Toc135747123)

[Table S15. Direct and indirect comparisons between interventions for Disease Activity outcome, as measured by Psoriasis Area and Severity Index (PASI)	52](#_Toc135747124)

[Safety secondary outcomes	53](#_Toc135747125)

[Total adverse events	53](#_Toc135747126)

[Table S16. League table of relative effects of interventions on the total adverse events outcome	53](#_Toc135747127)

[Figure S18. Network meta-analysis estimates the effect of interventions compared to placebo on the total adverse events	54](#_Toc135747128)

[Figure S19. Ranking of interventions based on their effect on the total adverse events outcome	55](#_Toc135747129)

[Certainty of evidence analysis	56](#_Toc135747130)

[Table S17. Certainty of evidence analysis of included studies in network meta-analysis for disease response outcome (ACR 50) at 6-month follow-up	56](#_Toc135747131)

[Table S18. Certainty of evidence analysis of included studies in network meta-analysis for disease response outcome (PsARC) at 6-month follow-up	58](#_Toc135747132)

[Table S19. Certainty of evidence analysis of included studies in network meta-analysis for serious adverse events outcome	60](#_Toc135747133)

Table S1. Search strategies performed in databases

| Databases (Search date) | Search Strategy | Nº of records identified |
| --- | --- | --- |
| Pubmed  (07/02/2022) | (((("Arthritis, Psoriatic"[Mesh]) OR (Psoria* Arthr*[Title/Abstract]))) AND (((("golimumab" [Supplementary Concept]) OR (Simponi[Title/Abstract])) OR ((((("Adalimumab"[Mesh]) OR (Humira[Title/Abstract])) OR (Amjevita[Title/Abstract])) OR (Cyltezo[Title/Abstract]))) OR (((("Etanercept"[Mesh]) OR (Erelzi[Title/Abstract])) OR (Enbrel[Title/Abstract]))) OR ((((("Infliximab"[Mesh]) OR (Inflectra[Title/Abstract])) OR (Remicade[Title/Abstract])) OR (Renflexis[Title/Abstract]))) OR (("secukinumab" [Supplementary Concept]) OR (Cosentyx[Title/Abstract]))) OR (("Certolizumab Pegol"[Mesh])) OR ((("tofacitinib" [Supplementary Concept]) OR (Tofacitinib*[Title/Abstract])) OR (Tasocitinib[Title/Abstract])) OR (Xeljanz[Title/Abstract]))) AND (((randomized controlled trial[pt] OR controlled clinical trial[pt] OR randomized[tiab] OR placebo[tiab] OR drug therapy[sh] OR randomly[tiab] OR trial[tiab] OR groups[tiab] NOT (animals [mh] NOT humans [mh])))) | 1950 |
| EMBASE (07/02/2022) | #1 'psoriatic arthritis'/exp OR 'psoria* arthr*':ti,ab  #2 'golimumab'/exp OR 'golimumab':ti,ab OR 'simponi*':ti,ab OR 'adalimumab'/exp OR 'humira':ti,ab OR 'adalimumab*':ti,ab OR 'etanercept'/exp OR 'enbrel':ti,ab OR 'etanercept*':ti,ab OR 'infliximab'/exp OR 'remicade':ti,ab OR 'infliximab*':ti,ab OR 'secukinumab'/exp OR 'cosentyx':ti,ab OR 'secukinumab':ti,ab OR 'certolizumab pegol'/exp OR 'certolizumab pegol':ti,ab OR 'cimzia':ti,ab OR 'tofacitinib'/exp OR 'xeljanz'  #3 'crossover procedure':de OR 'double-blind procedure':de OR 'randomized controlled trial':de OR 'single-blind procedure':de OR random*:de,ab,ti OR factorial*:de,ab,ti OR crossover*:de,ab,ti OR ((cross NEXT/1 over*):de,ab,ti) OR placebo*:de,ab,ti OR ((doubl* NEAR/1 blind*):de,ab,ti) OR ((singl* NEAR/1 blind*):de,ab,ti) OR assign*:de,ab,ti OR allocat*:de,ab,ti OR volunteer*:de,ab,ti  #4 (#1 AND #2 AND #3)  #5 AND [embase]/lim NOT ([embase]/lim AND [medline]/lim) | 1411 (Embase) |
| Cochrane Library – CENTRAL  (07/02/2022) | #1 MeSH descriptor: [Arthritis, Psoriatic] explode all trees 510  #2 Psoriat* Arthr* 2613  #3 #1 OR #2 2613  #4 Golimumab 800  #5 Simponi (:ti,ab,kw) 56  #6 MeSH descriptor: [Adalimumab] explode all trees 827  #7 Cyltezo (:ti,ab,kw) 3  #8 Humira (:ti,ab,kw) 458  #9 Amjevita (:ti,ab,kw) 4  #10 MeSH descriptor: [Etanercept] explode all trees 801  #11 Enbrel (:ti,ab,kw) 284  #12 MeSH descriptor: [Infliximab] explode all trees 780  #13 Inflectra (:ti,ab,kw) 25  #14 Renflexis (:ti,ab,kw) 2  #15 Remicade (:ti,ab,kw) 299  #16 Secukinumab 1005  #17 MeSH descriptor: [Certolizumab Pegol] explode all trees 184  #18 Cimzia (:ti,ab,kw) 60  #19 Tofacitinib 938  #20 Xeljanz (:ti,ab,kw) 25  #21 {OR #4-#20} 5581  #22 #3 AND #21 in Trials 825 | 825 |
| LILACS via BVS  (07/02/2022) | ((mh:(Artrite Psoriásica)) OR (mh:(Arthritis, Psoriatic)) OR (Artrite Psoriática) OR (Psoríase Artrítica) OR (Arthr$ Psoria$) OR (Psoria$ Arthr$) OR (Psoriasis Artr$) ) AND ((golimumabe) OR (golimumab) OR (simponi) OR (mh:(Adalimumab)) OR (Adalimumab$) OR (Humira) OR (Amjevita) OR (Cyltezo) OR (mh:(Etanercepte)) OR (mh:(Etanercept)) OR (Enbrel) OR (Etanercept$) OR (mh:(Infliximabe)) OR (mh:(Infliximab)) OR (Infliximab$) OR (Inflectra) OR (Remicade) OR (Renflexis) OR (Secuquinumabe) OR (Secukinumab) OR (Cosentyx) (mh:(Certolizumab Pegol)) OR (Cetolizumabe Pegol) OR (Cimzia) OR (Tofacitinibe) OR (Xeljanz)) | 60 |
| ClinicalTrials. Gov  (07/02/2022) | Golimumab OR Adalimumab OR Etanercept OR Infliximab OR Secukinumab OR tofacitinib \| Psoriasis, Arthritic | 143 |
| ICTRP  (07/02/2022) | ((Golimumab OR Adalimumab OR Etanercept OR Infliximab OR Secukinumab OR tofacitinib) AND Psoriatic arthritis) | 199 |

Table S2. List of excluded records (and the reason for exclusion) in the full-text eligibility analysis

| Study | Publication Title | Reason for exclusion |
| --- | --- | --- |
| Smolen 2020 | A head-to-head comparison of the efficacy and safety of ixekizumab and adalimumab in biological-naïve patients with active psoriatic arthritis: 24-week results of a randomised, open-label, blinded-assessor trial | Other comparator |
| Smolen 2020 | Multicentre, randomised, open-label, parallel-group study evaluating the efficacy and safety of ixekizumab versus adalimumab in patients with psoriatic arthritis naïve to biological disease-modifying antirheumatic drug: final results by week 52 | Other comparator |
| Smolen 2020 | Efficacy and Safety of Ixekizumab with or Without Methotrexate in Biologic-Naïve Patients with Psoriatic Arthritis: 52-Week Results from SPIRIT-H2H Study | Other comparator |
| Nash 2021 | Tofacitinib as monotherapy following methotrexate withdrawal in patients with psoriatic arthritis previously treated with open-label tofacitinib plus methotrexate: a randomised, placebo-controlled substudy of OPAL Balance | Other intervention |
| Raffayova 2009 | Infliximab plus methotrexate significantly improves synovitis and psoriatic lesions in methotrexate nave psoriatic arthritis (PsA) patients: results of the RESPOND trial | Multiple reports of the same study |
| Asahina 2016 | Oral tofacitinib efficacy, safety and tolerability in Japanese patients with moderate to severe plaque psoriasis and psoriatic arthritis: a randomized, double-blind, phase 3 study | Other comparator |
| Syversen 2020 | Therapeutic Drug Monitoring Compared to Standard Treatment of Patients Starting Infliximab: Results from a Multicenter Randomized Controlled Trial of 400 Patients | Insufficient data |
| Kavanaugh 2013 | Sustained improvements in productivity at paid work and within household, and increased participation in daily activities over time with certolizumab pegol in patients with psoriatic arthritis: 48-week results from the rapid-psa study | Insufficient data |
| Nct 2019 | Study to Demonstrate the Efficacy, Safety and Tolerability of Intravenous Secukinumab up to 52 Weeks in Subjects with Active Psoriatic Arthritis | Insufficient data |
| Euctr 2016 | Study of the efficacy and safety of secukinumab in patients with active psoriatic arthritis with axial skeleton involvement | Multiple reports of the same study |
| Nct 2016 | Study of Power Doppler Ultrasound (PDUS) to Measure Response of Secukinumab Treatment in Patients with Active Psoriatic Arthritis (PsA) | Multiple reports of the same study |
| McInnes 2020 | Secukinumab versus adalimumab head-tohead comparison in biologic-naïve patients with active psoriatic arthritis through 52-weeks (EXCEED): a randomised, doubleblind, phase-3b study | Insufficient data |
| Nash 2021 | Secukinumab provides sustained improvement in nail psoriasis, signs and symptoms of psoriatic arthritis and Low rate of radiographic progression in patients with concomitant nail involvement: 2-year results from the Phase III FUTURE 5 study | Multiple reports of the same study |
| Nash 2019 | Secukinumab provides improvement in nail psoriasis and inhibition of radiographic progression in psoriatic arthritis patients with nail phenotype: 52-week results from a phase iii study | Insufficient data |
| Mease 2018 | Secukinumab in the treatment of psoriatic arthritis: efficacy and safety results through 3 years from the year 1 extension of the randomised phase III FUTURE 1 trial | Other comparator |
| McInnes 2015 | Secukinumab, a human anti-interleukin-17A monoclonal antibody, in patients with psoriatic arthritis (FUTURE 2): a randomised, double-blind, placebo-controlled, phase 3 trial | Multiple reports of the same study |
| Mease 2016 | Safety and efficacy of ABT-122, a TNF and IL-17-targeted dual variable domain (DVD)-Ig™, in psoriatic arthritis patients with inadequate response to methotrexate: Results from a phase 2 trial | Insufficient data |
| Ramos 2016 | Reduced CLEC9A expression in synovial tissue of psoriatic arthritis patients after adalimumab therapy | Other outcome |
| Euctr 2010 | PHASE 3, MULTICENTER, RANDOMIZED, DOUBLE BLIND, PARALLEL GROUP, PLACEBO CONTROLLED STUDY TO EVALUATE THE EFFICACY AND SAFETY OF CERTOLIZUMAB PEGOL IN SUBJECTS WITH ADULT ONSET ACTIVE AND PROGRESSIVE PSORIATIC ARTHRITIS (PSA) | Multiple reports of the same study |
| Mease 2011 | Minimally important difference of Health Assessment Questionnaire in psoriatic arthritis: relating thresholds of improvement in functional ability to patient-rated importance and satisfaction | Multiple reports of the same study |
| Aalbers 2015 | Intra-articular etanercept treatment in inflammatory arthritis: a randomized double-blind placebo-controlled proof of mechanism clinical trial validating TNF as a potential therapeutic target for local treatment | População errada |
| Torii 2010 | Infliximab monotherapy in Japanese patients with moderate-to-severe plaque psoriasis and psoriatic arthritis. A randomized, double-blind, placebo-controlled multicenter trial | População errada |
| Euctr 2013 | Extension study up to three years for secukinumab in psoriatic arthritis | Multiple reports of the same study |
| Nct 2015 | Etanercept and Methotrexate in Combination or as Monotherapy in Psoriatic Arthritis | Multiple reports of the same study |
| Euctr 2013 | Efficacy at 24 weeks with long term safety, tolerability and efficacy up to 5 years of secukinumab (AIN457) in patients of active psoriatic arthritis (PsA) | Multiple reports of the same study |
| McInnes 2020 | Efficacy and Safety of Upadacitinib versus Placebo and Adalimumab in Patients with Active Psoriatic Arthritis and Inadequate Response to Non-Biologic Disease-Modifying Anti-Rheumatic Drugs: A Double-Blind, Randomized Controlled Phase 3 Trial | Insufficient data |
| Araujo 2019 | Effects of ustekinumab versus tumor necrosis factor inhibition on enthesitis: results from the enthesial clearance in psoriatic arthritis (ECLIPSA) study | Other study design |
| Rudwaleit 2010 | Effectiveness and safety of adalimumab in patients with ankylosing spondylitis or psoriatic arthritis and history of anti-tumor necrosis factor therapy | Other study design |
| FitzGerald 2017 | Certolizumab pegol is associated with long-term improvements in extra-articular manifestations of psoriatic arthritis over 4-years of treatment | Multiple reports of the same study |
| Nct 2010 | Certolizumab Pegol in Subjects with Adult Onset Active and Progressive Psoriatic Arthritis | Multiple reports of the same study |
| Euctr 2019 | A study to test the efficacy and safety of bimekizumab in the treatment of subjects with active psoriatic arthritis | Insufficient data |
| Nct 2005 | A Study of the Safety and Efficacy of Golimumab in Patients with Active Psoriatic Arthritis | Multiple reports of the same study |
| Euctr 2021 | A Study of the Efficacy and Safety of Guselkumab and Golimumab in Participants with Active Psoriatic Arthritis | Insufficient data |
| Euctr 2016 | A study of subjects with Psoriatic Arthritis to investigate the effectiveness of adalimumab introduction compared with methotrexate dose escalation | Multiple reports of the same study |
| Nct 2016 | A Study of Subjects with Psoriatic Arthritis to Investigate the Effectiveness of Adalimumab Introduction Compared With Methotrexate Dose Escalation (CONTROL) | Multiple reports of the same study |
| Nct 2017 | A Study of Ixekizumab (LY2439821) Versus Adalimumab in Participants with Psoriatic Arthritis | Multiple reports of the same study |
| Euctr 2014 | A Study of Golimumab in Participants with Active Psoriatic Arthritis | Multiple reports of the same study |
| Nct 2014 | A Study of Golimumab in Participants with Active Psoriatic Arthritis | Multiple reports of the same study |
| Euctr 2015 | A study comparing the use of etanercept and methotrexate, used either alone or in combination for the treatment of Psoriatic Arthritis | Multiple reports of the same study |
| Euctr 2005 | A randomized, multicenter, international, open-label study of infliximab plus methotrexate versus methotrexate (MTX) alone for the treatment of MTX naïve subjects with Active Psoriatic Arthritis | Multiple reports of the same study |
| Euctr 2013 | A randomized, double-blind, placebo-controlled trial of golimumab+methotrexate versus methotrexate alone in methotrexate-naïve patients with psoriatic arthritis | Insufficient data |
| Per 2012 | A randomized, double-blind, placebo-controlled, multicenter study of secukinumab to demostrate the efficacy at 24 weeks and to assess the safety, tolerability and long term efficacy up to 2 year in patients with active psoriatic arthritis | Insufficient data |
| Euctr 2013 | A phase 3 study of the efficacy and safety of 2 doses of tofacitinib (cp-690,550) or adalimumab in subjects with active psoriatic arthritis | Multiple reports of the same study |
| Euctr 2005 | A Multicenter, Randomized, Double-blind, Placebo-controlled Trial of Golimumab, a Fully Human Anti-TNFalfa Monoclonal Antibody, Administered Subcutaneously in Subjects with Active Psoriatic Arthritis - NA | Insufficient data |
| Euctr 2014 | 24 week efficacy and 3-year safety and efficacy of secukinumab in active Psoriatic Arthritis | Multiple reports of the same study |
| Nct 2013 | 24 Week Efficacy and 3-year Safety and Efficacy of Secukinumab in Active Psoriatic Arthritis | Multiple reports of the same study |
| Euctr 2011 | 2 year study of secukinumab (AIN457) treatment in patients with active Psoriatic Arthritis | Insufficient data |
| Nct 2014 | 16-week Efficacy and 2-year Safety, Tolerability and Efficacy of Secukinumab in Participants with Active Psoriatic Arthritis | Multiple reports of the same study |
| Mease 2019 | Etanercept and methotrexate as monotherapy or in combination in patients with psoriatic arthritis: a phase 3, double- blind, randomized controlled study | Insufficient data |
| D'Agostino 2021 | Towards development of an ultrasound enthesitis score in psoriatic arthritis: 24-week results from the phase iii randomised ultimate study | Insufficient data |
| VanDerHeijde 2017 | Tofacitinib treatment in patients with psoriatic arthritis and rates of radiologic progression according to baseline crp levels: results from a phase 3 clinical study | Insufficient data |
| Nash 2019 | Tofacitinib as monotherapy following methotrexate withdrawal in patients with psoriatic arthritis previously treated with open-label tofacitinib + methotrexate: a randomized, placebo-controlled sub-study of opal balance | Multiple reports of the same study |
| Nash 2020 | Tofacitinib as monotherapy following methotrexate withdrawal in patients with psoriatic arthritis previously treated with open-label tofacitinib + methotrexate: a randomised, placebo-controlled substudy of opal balance | Multiple reports of the same study |
| Nash 2019 | The impact of time since first diagnosis on the efficacy and safety of tofacitinib in patients with active psoriatic arthritis | Multiple reports of the same study |
| Merola 2019 | The impact of psoriasis severity on outcomes among psoriatic arthritis patients receiving Adalimumab | População errada |
| Strand 2020 | The impact of adalimumab vs placebo on patient-reported outcomes and utility measures among patients with moderately to severely active psoriatic arthritis | Insufficient data |
| Strand 2019 | The impact of adalimumab vs placebo on patient-reported outcomes and utility measures among patients with moderately to severely active Psoriatic Arthritis | Insufficient data |
| Khraishi 2017 | The effect of certolizumab pegol on skin manifestations of psoriatic arthritis over 4 years of treatment in patients with and without prior anti-TNF exposure | Multiple reports of the same study |
| Hodge 2017 | Switching from adalimumab to CHS-1420: A randomized, double-blind global clinical trial in patients with psoriasis and psoriatic arthritis | Insufficient data |
| deJong 2019 | Sustained remission with methotrexate monotherapy after 22-week induction treatment with TNF-alpha inhibitor and methotrexate in early psoriatic arthritis: an open-label extension of a randomized placebo-controlled trial | Other study design |
| Navarra 2018 | Subcutaneous secukinumab improves psoriatic arthritis symptoms and inhibits radiographic progression: Analysis by prior anti-TNF therapy from FUTURE-5 study | Multiple reports of the same study |
| Nct 2021 | Study of Efficacy and Safety of Secukinumab in Chinese Subjects with Active PsA Compared to Placebo | Multiple reports of the same study |
| D'Agostino 2021 | Secukinumab significantly decreased jointsynovitis measured by Power Dopplerultrasonography in biologic-naive patients withactive psoriatic arthritis: primary (12week) resultsfrom a randomised, placebo-controlled Phase 3 study | Insufficient data |
| D'Agostino 2020 | Secukinumab Significantly Decreased Joint Synovitis Measured by Power Doppler Ultrasonography in Biologic-naïve Patients with Active Psoriatic Arthritis: primary (12-Week) Results from a Randomized, Placebo-Controlled Phase III Study | Insufficient data |
| Coates 2018 | Secukinumab provides sustained pasdas related Low disease activity in psoriatic arthritis: Two year results from the future 2 study | Multiple reports of the same study |
| Hall 2017 | Secukinumab provides sustained improvements in the signs and symptoms of active psoriatic arthritis (PSA) through 3 years: efficacy and safety results from a phase 3 trial | Insufficient data |
| Nash 2019 | Secukinumab provides significant and sustained improvement in nail psoriasis and signs and symptoms of psoriatic arthritis in patients with nail phenotype: 52-week results from the phase iii future 5 study | Insufficient data |
| Kirkham 2015 | Secukinumab is effective in reducing dactylitis and enthesitis using multiple measures in patients with psoriatic arthritis: data from a phase 3 randomized, multicenter, double-blind, placebo-controlled study (future 2) | Multiple reports of the same study |
| VanDerHeijde 2015 | Secukinumab inhibits radiographic progression in patients with psoriatic arthritis: data from a phase 3 randomized, multicenter, double-blind, placebo-controlled study (future 1) | Multiple reports of the same study |
| Bissonnette 2018 | Secukinumab demonstrates high sustained efficacy and a favorable safety profile through five years of treatment in moderate-to-severe psoriasis | Multiple reports of the same study |
| Gu 2019 | Secukinumab 300 mg showed faster and higher efficacy in Chinese moderate to severe plaque psoriasis patients | População errada |
| Arendse 2018 | Safety and effectiveness of ustekinumab for the treatment of psoriatic arthritis over a 6 month period | Other study design |
| Arendse 2017 | Safety and effectiveness of ustekinumab for the treatment of psoriatic arthritis over a 6 month period | Other intervention |
| Kivitz 2016 | Randomized, double-blind study comparing CHS-0214 with etanercept (ENBREL) in patients with psoriasis and psoriatic arthritis | Other intervention |
| Mease 2004 | Pneumococcal vaccine response in psoriatic arthritis patients during treatment with etanercept | Other outcome |
| Deodhar 2019 | Integrated safety analysis across phase 3 clinical studies including the controlled and uncontrolled periods for intravenous golimumab in rheumatoid arthritis, psoriatic arthritis, and ankylosing spondylitis | Other study design |
| Euctr 2015 | GOLMePsA | Multiple reports of the same study |
| Vieira-Sousa 2019 | GO-DACT: a randomized double-blind placebocontroled proof-of-concept trial of golimumab plus methotrexate (MTX) versus MTX monotherapy, in improving dactylitis, in MTX naÃ¯ve psoriatic arthritis patients | Multiple reports of the same study |
| Behrens 2019 | Efficacy of ixekizumab in different phenotypes of patients with active psoriatic arthritis: Results from the SPIRIT trials | Multiple reports of the same study |
| McInnes 2020 | Efficacy and safety of upadacitinib versus placebo and adalimumab in patients with active psoriatic arthritis and inadequate response to non-biologic disease-modifying anti-rheumatic drugs (SELECT-PSA-1): A doubleblind, randomized controlled phase 3 trial | Multiple reports of the same study |
| Smolen 2021 | Efficacy and safety of ixekizumab versus adalimumab (SPIRIT-H2H) with and without concomitant conventional synthetic disease-modifying antirheumatic drugs (DMARD) in biologic dmard-naïve patients with psoriatic arthritis: 52-week results | Insufficient data |
| Damjanov 2018 | Efficacy and safety of etanercept in psoriasis and psoriatic arthritis in the PRESTA study: analysis in patients from Central and Eastern Europe | Other comparator |
| Husni 2017 | Effects of intravenous golimumab, an anti-TNFα monoclonal antibody, on mental and physical functioning and health-related quality of life in active psoriatic arthritis: 24-week results of a phase 3 trial | Insufficient data |
| Goupille 2021 | Effect of secukinumab versus adalimumab on acr core components and health-related quality of life in patients with psoriatic arthritis: results from the exceed study | Insufficient data |
| Coates 2016 | Effect of concomitant conventional disease-modifying antirheumatic drugs (cDMARDs) on the efficacy and safety of ixekizumab in biologic dmard-naive patients with active psoriatic arthritis | Insufficient data |
| Nct 2006 | Double-blind, Randomized, Placebo-controlled Phase 3 Study of Etanercept in the Treatment of Psoriatic Arthritis and Psoriasis | Multiple reports of the same study |
| Kivitz 2018 | Arthritis: primary results through 52weeks from a phase-3 randomized placebo-controlled study (future 4) | Multiple reports of the same study |
| Bolt 2021 | Adalimumab has no significant effect on interleukin-17 and interleukin-17 receptor expression in skin and synovium of psoriatic arthritis patients with mild psoriasis | Other study design |
| Euctr 2017 | A Study Comparing ABT-494 to Placebo and to Adalimumab in Subjects with Psoriatic Arthritis Who Have an Inadequate Response to at Least One Non-Biologic Disease Modifying Anti-Rheumatic Drug | Multiple reports of the same study |
| Euctr 2010 | A Phase 3 Multicenter, Randomized, Double-blind, Placebo-controlled trial of Ustekinumab, a Fully Human anti-IL-12/23p40 Monoclonal Antibody, Administered Subcutaneously, in Subjects with Active Psoriatic Arthritis Including Those Previously Treated with Biologic Anti-TNFa Agent(s) - PSUMMIT II | Other intervention |
| Euctr 2015 | Study to demonstrate the efficacy (including inhibition of structural damage), safety and tolerability up to 2 years of secukinumab in Active Psoriatic Arthritis | Multiple reports of the same study |
| Euctr 2013 | A study of the efficacy and safety of 2 doses of tofacitinib (cp-690,550) in subjects with active psoriatic arthritis and an inadequate response to at least one tnf inhibitor | Multiple reports of the same study |
| Raffayova 2009 | Infliximab plus methotrexate significantly improves rates of remission for methotrexate nave psoriatic arthritis (PsA) patients compared to methotrexate alone: The RESPOND trial | Multiple reports of the same study |
| D 2010 | Golimumab, a new, human, TNF alpha antibody, administered subcutaneously every 4 weeks in psoriatic arthritis patients: 104-week efficacy and safety results of the randomized, placebo-controlled go-reveal study | Multiple reports of the same study |
| Furst 2013 | The effect of golimumab on haemoglobin levels in patients with rheumatoid arthritis, psoriatic arthritis or ankylosing spondylitis | Other study design |
| Nct 2015 | Study to Demonstrate the Efficacy (Including Inhibition of Structural Damage), Safety and Tolerability up to 2 Years of Secukinumab in Active Psoriatic Arthritis | Multiple reports of the same study |
| Bissonnette 2017 | Secukinumab demonstrates sustained high efficacy and a favorable safety profile in moderate to severe psoriasis patients through 4-years of treatment (extension of the SCULPTURE study) | Multiple reports of the same study |
| Husni 2018 | Intravenous golimumab in adult patients with active psoriatic arthritis: efficacy and safety through 1 year | Multiple reports of the same study |
| Kavanaugh 2016 | Efficacy of Subcutaneous Secukinumab in Patients with Active Psoriatic Arthritis Stratified by Prior Tumor Necrosis Factor Inhibitor Use: results from the Randomized Placebo-controlled FUTURE 2 Study | Multiple reports of the same study |
| McInnes 2015 | Secukinumab improves signs and symptoms of active psoriatic arthritis in a phase 3 randomized, multicenter, double-blind, placebo-controlled study using a subcutaneous dosing regimen (future 2) | Multiple reports of the same study |
| Baraliakos 2020 | Secukinumab improves clinical and imaging outcomes in patients with psoriatic arthritis and axial manifestations with inadequate response to NSAIDs: week 52 results from the maximise trial | Multiple reports of the same study |
| Baraliakos 2019 | Secukinumab improves axial manifestations in patients with psoriatic arthritis and inadequate response to nsaids: primary analysis of phase 3 trial | Multiple reports of the same study |
| Freundlich 2009 | Results of a randomized, double-blind study to evaluate the efficacy and safety of etanercept in patients with psoriasis and psoriasis and psoriatic arthritis: The presta trial | Multiple reports of the same study |
| Nct 2006 | Remicade Study in Psoriatic Arthritis Patients of Methotrexate-Naïve Disease (RESPOND) (Study P04422) | Multiple reports of the same study |
| Nct 2014 | Efficacy of Golimumab in Combination with Methotrexate (Mtx) Versus Mtx Monotherapy, in Improving Dactylitis, in Mtx naïve Psoriatic Arthritis Patients | Multiple reports of the same study |
| VanDerHeijde 2018 | Radiographic progression of structural joint damage in patients with active psoriatic arthritis treated with ixekizumab for up to 3 years | Multiple reports of the same study |
| Nct 2018 | Safety and Efficacy of Tofacitinib vs Methotrexate in the Treatment of Psoriatic Arthritis | Insufficient data |
| Nct 2010 | A Study of the Safety and Efficacy of Ustekinumab in Patients With Psoriatic Arthritis With and Without Prior Exposure to Anti-TNF Agents | Other intervention |
| McInnes 2011 | Anti-interleukin 17A monoclonal antibody secukinumab reduces signs and symptoms of psoriatic arthritis in a 24-week multicenter, double-blind, randomized, placebo-controlled trial | Multiple reports of the same study |
| Raju 2009 | Assessing HRQoL burden of disease and comparison to the U.S. population in psoriatic arthritis patients treated with golimumab: Results from the GO-REVEAL trial | Multiple reports of the same study |
| Merola 2019 | Improvement in the signs and symptoms of psoriatic arthritis with ixekizumab compared to adalimumab in patient subgroups defined by baseline disease characteristics | Insufficient data |
| Coates 2016 | Ixekizumab provides sustained improvement up to 52 weeks of disease activity as assessed by composite measure scores in biologic disease-modifying antirheumatic drug-naive patients with active psoriatic arthritis | Insufficient data |
| Kavanaugh 2015 | Secukinumab efficacy in anti-TNF-naive and anti-TNF-IR patients with psoriatic arthritis: results of a phase 3 multicenter, double-blind, placebo-controlled study (future 2) | Multiple reports of the same study |
| VanDerHeijde 2014 | Secukinumab, a monoclonal antibody to interleukin-17a, provides significant and sustained inhibition of joint structural damage in active psoriatic arthritis regardless of prior TNF inhibitors or concomitant methotrexate: A phase 3 randomized, double-blind, placebo-controlled study | Insufficient data |
| Husni 2019 | Improvements in health-related quality of life in psoriatic arthritis patients treated with intravenous golimumab, an anti-TNFα monoclonal antibody: 1-year results of a phase III trial | Multiple reports of the same study |
| Husni 2020 | Improvements in health-relatedqualityof life in psoriatic arthritis patients treatedwith intravenousgolimumab,ananti-tnfαmonoclonal antibody: 1-year results ofa phase iii trial | Insufficient data |
| Rahman 2010 | Golimumab inhibits progression of radiographic damage in patients with psoriatic arthritis: 52 week results from the go-reveal study | Multiple reports of the same study |
| Aalbers 2012 | Intra-articular etanercept treatment in inflammatory arthritis: A randomized double-blind placebo-controlled trial | População errada |
| Burkhardt 2016 | A randomized, double-blind, active-and placebo (PBO)controlled phase 3 study of efficacy and safety of ixekizumab (IXE), adalimumab (ADA), and placebo therapy in patients na- ive to biologic disease-modifying antirheumatic drugs (bDMARDs) with active psoriatic arthritis (PsA) | Insufficient data |
| Gladman 2017 | Efficacy and safety of tofacitinib, an oral Janus kinase inhibitor, in patients with active psoriatic arthritis and an inadequate response to tumor necrosis factor inhibitors: OPAL Beyond, a randomized, double blind, placebo-controlled, phase 3 trial | Multiple reports of the same study |
| Husni 2018 | Clinically meaningful improvement in skin and nail psoriasis in bio-naïve active psoriatic arthritis patients treated with intravenous golimumab: results through week 24 from a phase 3 study | Multiple reports of the same study |
| Smolen 2019 | A head-to-head comparison of ixekizumab and adalimumab in biologic-naïve patients with active psoriatic arthritis: Efficacy and safety outcomes from a randomized, open-label, blinded assessor study through 52 weeks | Multiple reports of the same study |
| Merola 2020 | 13350 Psoriasis outcomes in a randomized trial of etanercept and methotrexate as monotherapy or in combination in patients with psoriatic arthritis | Insufficient data |
| Krueger 2013 | Effects of golimumab on the dermatologic manifestations of psoriatic arthritis: 5-year results from the long-term extension of the randomized, placebo-controlled, GO-REVEAL study | Multiple reports of the same study |
| Atzeni 2011 | Etanercept plus ciclosporin versus etanercept plus methotrexate for maintaining clinical control over psoriatic arthritis: a randomised pilot study | Other intervention |
| Brodszky 2008 | Efficacy of adalimumab, etanercept, and infliximab in psoriatic arthritis based on ACR50 response after 24 weeks of treatment | Other study design |
| Blauvelt 2014 | Secukinumab efficacy in subjects with moderate-to-severe plaque psoriasis and concomitant psoriatic arthritis: a subanalysis of the ERASURE study | Other study design |
| Husni 2017 | Impact of golimumab, an anti-TNFa monoclonal antibody, on health related quality of life (HRQOL) and work productivity in patients with active psoriatic arthritis: 24-week results of the phase III go vibrant trial | Insufficient data |
| Gladman 2016 | Efficacy and safety of tofacitinib, an oral janus kinase inhibitor, in patients with active psoriatic arthritis and an inadequate response to tumor necrosis factor inhibitors: opal beyond, a randomized, double blind, placebo-controlled, phase 3 trial | Multiple reports of the same study |
| Baraliakos 2019 | Secukinumab improves axial manifestations in patients with psoriatic arthritis and inadequate response to NSAIDs: primary analysis of the MAXIMISE trial | Multiple reports of the same study |
| Baraliakos 2020 | Secukinumab improves axial manifestations in psoriatic arthritis patients with inadequate response to nsaids: primaryanalysis of phase iii maximise trial | Insufficient data |
| Boggs 2014 | Employment is maintained and sick days decreased in psoriasis/psoriatic arthritis patients with etanercept treatment | Other intervention |
| Mease 2016 | Efficacy and safety of tofacitinib, an oral janus kinase inhibitor, or adalimumab in patients with active psoriatic arthritis and an inadequate response to conventional synthetic DMARDS: a randomized, placebo-controlled, phase 3 trial | Multiple reports of the same study |
| Boers 2021 | Responsiveness of ultrasound synovitis and clinical outcomes in psoriatic arthritis treated with secukinumab: Data from the ultimate trial | Multiple reports of the same study |
| Nash 2015 | Secukinumab, a human anti-interleukin-17a monoclonal antibody, improves active psoriatic arthritis and inhibits radiographic progression: efficacy and safety data from a phase 3 randomized, multicenter, double-blind, placebo-controlled study | Insufficient data |
| Nash 2015 | Secukinumab, a human anti-interleukin-17a monoclonal antibody, significantly reduces psoriasis burden in patients with psoriatic arthritis: results from a phase 3 randomized controlled trial | Multiple reports of the same study |
| McInnes 2012 | Secukinumab, a fully human anti-interleukin-17A antibody, improves signs and symptoms of psoriatic arthritis: A 24-week, double-blind, placebocontrolled, multicenter trial | Insufficient data |
| Coates 2016 | Ixekizumab provides sustained improvement up to 52 weeks of disease activity as assessed by composite measure scores in biologic diseasemodifying antirheumatic drug (BDMARD)-naive patients with active psoriatic arthritis | Insufficient data |
| Kavanaugh 2013 | Improvements in productivity at paid work and within household, and increased participation in daily activities after 24 weeks of certolizumab pegol treatment of patients with psoriatic arthritis: Results of a phase 3 double blind randomized placebo-controlled study | Insufficient data |
| Husni 2019 | Clinically meaningful improvement in skin and nail psoriasis in bio-naÃ¯ve active psoriatic arthritis patients treated with intravenous golimumab: results through week 52 from a phase-3 study | Multiple reports of the same study |
| Coates 2016 | Effect of concomitant conventional disease-modifying antirheumatic drug (cDMARDS) on safety and efficacy of ixekizumab in biologic dmard (bDMARD) naive patients with active psoriatic arthritis (PsA) | Insufficient data |
| Cohen 2019 | Decreased Injection Site Pain Associated with Phosphate-Free Etanercept Formulation in Rheumatoid Arthritis or Psoriatic Arthritis Patients: A Randomized Controlled Trial | Other intervention |
| Prinz 2011 | Combination of skin, joint and quality of life outcomes with etanercept in psoriasis and psoriatic arthritis in the PRESTA trial | Other intervention |
| Nash 2017 | CONGRESS: APLAR 2017 Abstract topic 10: psoriatic arthritis Presentation type: oral/Poster Secukinumab leads to 2-year sustained minimal disease activity in patients with active psoriatic arthritis: results of the Phase-3 FUTURE-2 Study | Insufficient data |
| Sterry 2010 | Comparison of two etanercept regimens for treatment of psoriasis and psoriatic arthritis: PRESTA randomised double blind multicentre trial | Other intervention |
| Puig 2015 | Cardiometabolic profile, clinical features, quality of life and treatment outcomes in patients with moderate-to-severe psoriasis and psoriatic arthritis | Other intervention |
| VanMens 2018 | Achieving remission in psoriatic arthritis by early initiation of TNF inhibition: a double-blind, randomized, placebo-controlled trial of golimumab + methotrexate versus placebo + methotrexate | Insufficient data |
| Coates 2020 | Achievement of very Low disease activity and remission treatment targets is associated with reduced radiographic progression in patients with psoriatic arthritis treated with certolizumab pegol | Multiple reports of the same study |
| Nct 2019 | GOLimumab and Methotrexate Versus Methotrexate in Very Early PsA (GOLMEPsA) | Insufficient data |
| Mease 2013 | Effect of adalimumab on physical function, health-related quality of life, and work productivity in patients with peripheral spondyloarthritis: Results from the ability-2 clinical trial | População errada |
| Nct 2021 | An Efficacy Study of Secukinumab In Enthesitis of Psoriatic Arthritis Patients | Insufficient data |

Table S3. Characteristics of randomized clinical trials included in systematic review

| Study | Study name | Sample Size | Multi-center | Country in which the study conducted | Follow-up (weeks) | Study funding sources |
| --- | --- | --- | --- | --- | --- | --- |
| Antoni 2005a | IMPACT | 104 | Yes | United States; Canada; Other: 9 centers in Europe | 16 | Janssen Biotech, Inc., Biotech, Inc. |
| Antoni 2005b | IMPACT-2 | 200 | Yes | United States; Canada; Other: Europe | 24 | Janssen Biotech, Inc |
| Atteno 2010 | - | 100 | No | Other: Italy | 48 | No funding |
| Baraliakos 2021 | MAXIMISE | 498 | Yes | UK; Other: Belgium, Bulgaria, Czechia, Estonia, Finland, France, Germany, Greece, Hungary, Ireland, Israel, Italy, Poland, Romania, Russia, Spain, Switzerland | 12 | Novartis |
| Baranauskaite 2012 | RESPOND | 115 | Yes | Other: 25 centres in Europe, the Middle East, South Africa, and Turkey. | 16 | Schering-Plough Corporation, now Merck, Sharp & Dohme Corporation, Whitehouse Station, New Jersey, USA. |
| Coates 2020 | CONTROL | 246 | Yes | United States; UK; Canada; Australia; Other: Brazil, Bulgaria, Colombia, Czechia, Germany, Italy, Poland, Puerto Rico, Qatar, Spain, | 16 | AbbVie |
| D’Agostino 2021 | ULTIMATE | 166 | Yes | United States; UK; Canada; Netherland; Norway; Other: Argentina, Austria, Belgium, Colombia, Czechia, France, Germany, Hungary, Ireland, Italy, Mexico, Spain | 12 | Novartis Pharma AG, Basel, Switzerland. |
| deGroot 2012 | - | 22 | No | Netherland | 4 | Abbott B.V. |
| EUCTR2016-000972-91-BG 2016 | ACHILLES | 204 | Yes | UK; Other: Bulgaria, Czechia, Germany, Greece, Italy, Slovakia, Spain, | 24 | Novartis Pharmaceuticals |
| EUCTR2019-001996-35-HU 2020 | PENGUIN 1 | 67 | Yes | United States; Canada; Japan; Australia; Other: Bulgaria, Czechia, Hungary, Korea, New Zealand, Poland, Russian Federation, Spain, Taiwan | 16 | Gilead Sciences |
| Genovese 2007 | - | 102 | Yes | United States; Canada | 12 | Abbott Laboratories |
| Gladman 2017 | OPAL Beyond | 395 | Yes | United States; UK; Australia; Other: Belgium, Brazil, Czechia, France, Germany, Mexico, Poland, Russia, Slovakia, Spain, Taiwan, | 12 | Pfizer |
| Kavanaugh 2009 | GO REVEAL | 405 | Yes | United States; UK; Canada; Other: Belgium, Poland, Spain | 24 | Centocor Research and Development, Inc. and Schering-Plough |
| Kavanaugh 2017 |  | 480 | Yes | United States; Canada; Other: Belarus, Germany, Hungary, Lithuania, Poland, Romania, Russia, Spain, Ukraine | 24 | Janssen Research & Development, LLC |
| Kivitz 2019 | FUTURE 4 | 341 | Yes | United States; UK; Canada; Australia; Other: Belgium, Bulgaria, Czech Republic, France, Germany, Italy, Poland, Russian Federation, Sweden | 16 | Novartis |
| McInnes 2014 | - | 42 | Yes | UK; Netherland; Other: Germany | 24 | Novartis |
| McInnes 2015 | FUTURE 2 | 397 | Yes | United States; UK; Canada; Australia; Other: Belgium, Czechia, Germany, Poland, Puerto Rico, Russia, Thailand | 24 | Novartis |
| McInnes 2020 | EXCEED | 853 | Yes | United States; UK; Canada; Netherland; Denmark; Australia; Other: Bulgaria, Czechia, Estonia, Finland, France, Germany, Greece, Hungary, Iceland, India, Israel, Italy, Korea, Latvia, Lithuania, Poland, Portugal, Russia, Slovakia, Spain | 52 | Novartis |
| McInnes 2021 | Select-PsA 1 | 1705 | Yes | United States; UK; Canada; Netherland; Norway; China; Japan; Australia; Other: Argentina, Belarus, Belgium, Bosnia, Brazil, Bulgaria, Chile, Colombia, Croatia, Czechia, Estonia, Germany, Greece, Hong Kong, Hungary, Ireland, Israel, Italy, Korea, Latvia, Lithuania, Malaysia, Mexico, New Zealand, Poland, Portugal, Puerto Rico, Russia, Serbia, Singapore, Slovakia, Slovenia, South Africa, Spain, Switzerland, Taiwan, Turkey, Ukraine, | 24 | AbbVie |
| Mease 2000 | - | 60 | No | United States | 12 | Immunex Corporation |
| Mease 2004 | - | 205 | Yes | United States | 24 | Immunex Corporation |
| Mease 2005 | ADEPT | 315 | Yes | United States; UK; Canada; Other: Austria, Belgium, France, Germany and Italy | 24 | Abbott Laboratories |
| Mease 2014 | RAPID-PsA | 409 | Yes | United States; UK; Canada; Other: Argentina Belgium Brazil Czechia France Germany Hungary Ireland Mexico Poland Spain | 24 | UCB Pharm |
| Mease 2015 | FUTURE 1 | 606 | Yes | United States; UK; Canada; Australia; Other: Argentina, Belgium, Brazil, Bulgaria, Czech Republic, Germany, Israel, Italy, Philippines, Poland, Romania, Russian Federation, Singapore, Slovakia, Thailand | 24 | Novartis Pharmaceuticals |
| Mease 2017a | SPIRIT P-1 | 417 | Yes | United States; UK; Canada; Netherland; Japan; Other: Belgium, Bulgaria, Czechia, Estonia, France, Mexico, Poland, Russia, Spain, Ukraine, | 16 | Eli Lilly and Company |
| Mease 2017b | OPAL BROADEN | 422 | Yes | United States; UK; Canada; Australia; Other: Belgium, Bulgaria, Czechia, France, Germany, Hungary, Mexico, Poland, Russian Federation, Slovakia, Spain, Taiwan | 12 | Pfizer |
| Mease 2018 | FUTURE 5 | 996 | Yes | United States; UK; Canada; Netherland; Denmark; Other: Argentina, Austria, Chile, Czechia, Estonia, Finland, Germany, Greece, Guatemala, Hungary, India, Iceland, Israel, Italy, Latvia, Lithuania, Mexico, Philippines, Russia, Spain, Sweden, Thailand, Vietnam | 16 | Novartis |
| Mease 2019 | SEAM-PsA | 851 | Yes | United States; UK; Canada; Other: Argentina, Bulgaria, Chile, Czechia, France, Greece, Hungary, Latvia, Mexico, Poland, Portugal, Puerto Rico, Russia, South Africa, Spain | 24 | Amgen Inc. |
| Nash 2018 | FUTURE 3 | 414 | Yes | United States; UK; Canada; Netherland; Australia; Other: Bulgaria, Czechia, Germany, Italy, Puerto Rico, Russian Federation, Spain, Switzerland | 24 | Novartis |
| Nguyen 2019 | CHOICE | 258 | Yes | United States | 16 | Novartis |
| van Kuijk 2009 | - | 24 | No | Netherland | 4 | Abbott BV, The Netherlands; Academisch Medisch Centrum (AMC) (Netherlands) |
| vanMens 2019 | - | 51 | Yes | Netherland | 22 | MSD |
| Vieira-Sousa 2020 | GO-DACT | 44 | Yes | Other: Portugal | 24 | No funding |

Table S4. Population characteristics of randomized clinical trials included in systematic review

| Study | Population description | Age | Duration of symptoms (years) | Severity | Previous biologic treatment (Yes / No) | Gender (% females) | Body weight (kg) | Patients with axial or enthesitis (%) | Patients with peripheral arthritis (including dactylitis) (%) |
| --- | --- | --- | --- | --- | --- | --- | --- | --- | --- |
| Antoni 2005a | Patients (ages 18 years and older) with an established diagnosis of PsA of 6 months duration or longer | 45.45 | 11.35 | NR | Yes | 42.3 | NR | 25 | 49.05 |
| Antoni 2005b | Adult patients with active PsA diagnosed at least 6 months before the first infusion of study drug | 46.80 | 7.95 | NR | Yes | 39 | NR | 38.5 | 40.5 |
| Atteno 2010 | Patients with established PsA who experienced an inadequate response to a previous DMARD therapy | 48.50 | 6.67 | NR | Yes | 60 | NR | NR | NR |
| Baraliakos 2021 | Adult patients with PsA | 46.57 | 3 | NR | No | 50.61 | NR | 100 | 81.49 |
| Baranauskaite 2012 | Methotrexate-naive subjects with active polyarticular PsA | 41.21 | 3.25 | NR | No | 45.29 | NR | NR | NR |
| Coates 2020 | Adult patients with PsA naive to DMARD | 50.10 | NR | NR | No | 50 | NR | 81 | 57 |
| D'Agostino 2021 | Patients with active PsA who failed conventional synthetic DMARDs (csDMARDs) therapy and were naıve to biological DMARDs (bDMARDs). | 47.00 | 4 | NR | No | 55 | NR | 100 | NR |
| deGroot 2012 | Adult patients with active psoriatic arthritis | 45.25 | 14.95 | NR | NR | 36.35 | NR | NR | NR |
| EUCTR2016-000972-91-BG 2016 | Adult patient with PsA | 47.75 | 5.33 | NR | Yes | 55.4 | 84.99 | 64 | 0 |
| EUCTR2019-001996-35-HU 2020 | Adult patients with active psoriatic arthritis who are naive to biologic disease-modifying anti-rheumatic drug (DMARD) therapy | 47.93 | NR | NR | No | 55.4 | NR | NR | NR |
| Genovese 2007 | Adult patients with moderate to severe PsA | 49.05 | 7.35 | Moderate to severe | Yes | 46.05 | 90 | NR | NR |
| Gladman 2017 | Patients with active psoriatic arthritis who had previously had an inadequate response to tumor necrosis factor (TNF) inhibitors | 49.94 | 9.37 | NR | Yes | 55.34 | 85.7 | 69.68 | 49 |
| Kavanaugh 2009 | Adult patients with active PsA | 46.96 | 7.49 | NR | NR | 39.72 | NR | 77.28 | 34 |
| Kavanaugh 2017 | Adult patients with active PsA | 46.00 | 5.8 | NR | No | 48 | NR | 76.25 | 53.75 |
| Kivitz 2019 | Adult patients with active PsA | 49.06 | 6.07 | NR | Yes | 58.08 | 85.13 | 63.35 | 35.77 |
| McInnes 2014 | Adult patients with moderate to severe PsA | 47 | 6 | Moderate to severe | Yes | 64 | NR | NR | NR |
| McInnes 2015 | Adult patients with active PsA | 47.96 | NR | NR | Yes | 51.71 | 87.11 | 63.73 | 34.79 |
| McInnes 2020 | Adult patients with active PsA | 49 | 5.4 | NR | No | 49 | 83.8 | 58 | 31 |
| McInnes 2021 | Adult patients with active PsA who have an inadequate response to nonbiologic disease-modifying antirheumatic drugs | 50.9 | 6.05 | NR | Yes | 50.81 | NR | 59.42 | 29.7 |
| Mease 2000 | Adult patients with active PsA | 45 | 10 | NR | Yes | 43.5 | 86.05 | 86.05 | NR |
| Mease 2004 | Adult patients with active PsA | 47.45 | 9.1 | NR | NR | 49.09 | NR | NR | NR |
| Mease 2005 | Adults who had moderately to severely active PsA | 49.00 | NR | Moderate to severe | No | 44.42 | 85.74 | 37.46 | 37.14 |
| Mease 2014 | Adult patients with a diagnosis of PsA | 47.54 | 8.54 |  |  | 55.26 | 84.41 | 64.3 | 26.39 |
| Mease 2015 | Adult patients with active PsA | 48.97 | NR | NR | Yes | 54.47 | 82.9 | 61.4 | 53.5 |
| Mease 2017a | Adult patients with active PsA not previously treated with biologic agents | 49.62 | 6.59 | NR | No | 52.16 | NR | 54.58 | 29.97 |
| Mease 2017b | Patients with active psoriatic arthritis who previously had an inadequate response to conventional synthetic disease modifying antirheumatic drugs (DMARDs) | 47.86 | 6.11 | NR | Yes | 53.22 | NR | 66.54 | 56.25 |
| Mease 2018 | Adult patients with active PsA | 48.8 | 6.6 | Moderate to severe | Yes | 49.8 | 83.4 | 60.45 | 39.1 |
| Mease 2019 | Methotrexate-naïve adult patients early in the course of their PsA disease | 48.4 | 3.2 | NR | No | 50.8 | NR | 67.7 | 33.4 |
| Nash 2018 | Adult patients with active PsA | 49.83 | 7.54 | NR | Yes | 54.82 | 85.61 | 67.85 | 28.52 |
| Nguyen 2019 | US-only population of biologic-naive patients with moderate to severe active psoriatic arthritis | 51.9 | 3.5 | Moderate to severe | NR | 48.81 | 91.57 | 73.34 | 48.1 |
| van Kuijk 2009 | Adult patients with active PsA | 45 | 6.95 | NR | NR | 37.5 | NR | NR | NR |
| vanMens 2019 | Patients aged 18-70 years with PsA who are naive to MTX and TNFi | 46.67 | 0.5 | NR | No | 25.49 | NR | 21.57 | 33.33 |
| Vieira-Sousa 2020 | Patients with PsA along with active dactylitis and naive to MTX and biologic disease-modifying antirheumatic drugs (bDMARDs) | 45.7 | 3.9 | NR | No | 16 | NR | 52.3 | 100 |

Abbreviations: NR: not reported

Table S5. Interventions characteristics of randomized clinical trials included in systematic review

| **Study ID** | **Randomized** | **Interventions** | **Administration** | **Dosage (mg)** | **Frequency** | **Concomitant MTX** | **Outcomes** |
| --- | --- | --- | --- | --- | --- | --- | --- |
| Antoni 2005a | 52 | Infliximab | IV | 5 mg/kg | At weeks 0, 2, 6, and 14 | No | ACR 50; PsARC; Function (HAQ-DI continuous); Disease activity (DAPSA, DAS28, CDAI or MDA) as continuous outcome; Disease activity (DAPSA, DAS28, CDAI or MDA) as dichotomous outcome; Pain; Skin disease (PASI continuous); Skin disease (PASI dichotomous); Serious adverse events; Total adverse events |
|  | 52 | Placebo | IV | NA | At weeks 0, 2, 6, and 14 |  |  |
| Antoni 2005b | 100 | Infliximab | IV | 5 mg/kg | At weeks 0, 2, and 6 followed by maintenance dosing at weeks 14 and 22. | No | ACR 50; PsARC; Function (HAQ-DI continuous); Function (HAQ-DI dichotomous); Pain; Quality of life; Skin disease (PASI dichotomous) |
|  | 100 | Placebo | IV | NA | At weeks 0, 2, and 6 followed by maintenance dosing at weeks 14 and 22. |  |  |
| Atteno 2010 | 34 | Adalimumab | NR | 40 | Every other week | No | Function (HAQ-DI continuous); Skin disease (PASI continuous); Serious adverse events; Total adverse events |
|  | 36 | Etanercept | NR | 25 | Twice weekly |  |  |
|  | 30 | Infliximab | NR | 5 mg/kg | Every 6–8 weeks |  |  |
| Baraliakos 2021 | 165 | Secukinumab | SC | 150 | Weekly for 4 weeks and every 4 weeks there-after | No | Function (HAQ-DI continuous); Pain; Serious adverse events; Total adverse events |
|  | 167 | Secukinumab | SC | 300 | Weekly for 4 weeks and every 4 weeks there-after |  |  |
|  | 166 | Placebo | SC | NA | Weekly for 4 weeks and every 4 weeks there-after |  |  |
| Baranauskaite 2012 | 57 | Infliximab | IV | 5 | At weeks 0, 2, 6 and 14 | Yes | ACR 50; Function (HAQ-DI continuous); Disease activity (DAPSA, DAS28, CDAI or MDA) as continuous outcome; Disease activity (DAPSA, DAS28, CDAI or MDA) as dichotomous outcome; Pain; Skin disease (PASI continuous); Skin disease (PASI dichotomous); Serious adverse events; Total adverse events |
|  | 58 | No treatment | NA | NA | NA |  |  |
| Coates 2020 | 123 | Adalimumab | SC | 40 | Every other week | Yes | ACR 50; Function (HAQ-DI continuous); Function (HAQ-DI dichotomous); Disease activity (DAPSA, DAS28, CDAI or MDA) as continuous outcome; Disease activity (DAPSA, DAS28, CDAI or MDA) as dichotomous outcome; Quality of life; Skin disease (PASI dichotomous); Serious adverse events; Total adverse events |
|  | 122 | No treatment | NA | NA | NA |  |  |
| D'Agostino 2021 | 83 | Secukinumab | SC | 150 or 300 | Weekly followed by 4-weekly dosing at Weeks 4 and 8 | No | ACR 50; Function (HAQ-DI continuous); Serious adverse events; Total adverse events |
|  | 83 | Placebo | SC | NA | Weekly followed by 4-weekly dosing at Weeks 4 and 8 |  |  |
| deGroot 2012 | 11 | Adalimumab | SC | 40 | A baseline and a second injection 2 weeks later | No | Skin disease (PASI continuous) |
|  | 11 | Placebo | SC | NA | At baseline and a second injection 2 weeks later |  |  |
| EUCTR2016-000972-91-BG 2016 | 64 | Secukinumab | SC | 150 or 300 | Week 0,1,2,3 150 mg or 300 mg Secukinumab s.c. Maintenance: 150 mg or 300 mg Secukinumab s.c. every 4 weeks starting at Week 4 | No | Pain; Quality of life |
|  | 64 | Placebo | SC | NA | Week 0,1,2,3 Secukinumab Placebo s.c. Maintenance: Secukinumab Placebo s.c. every 4 weeks starting at Week 4 until Week 24 followed by 150 or 300 mg Secukinumab s.c. every 4 weeks |  |  |
| EUCTR2019-001996-35-HU 2020 | 9 | Adalimumab | SC | 40 | Once every 2 weeks | No | ACR 50; PsARC; Function (HAQ-DI continuous); Disease activity (DAPSA, DAS28, CDAI or MDA) as continuous outcome; Disease activity (DAPSA, DAS28, CDAI or MDA) as dichotomous outcome; Quality of life; Skin disease (PASI continuous); Skin disease (PASI dichotomous); Serious adverse events; Total adverse events |
|  | 20 | Placebo | SC | NA | Once every 2 weeks |  |  |
| Genovese 2007 | 51 | Adalimumab | SC | 40 | Every other week | No | ACR 50; PsARC; Function (HAQ-DI continuous); Pain; Quality of life; Serious adverse events; Total adverse events |
|  | 51 | Placebo | SC | NA | Every other week |  |  |
| Gladman 2017 | 131 | Tofacitinib | Oral | 5 | Twice daily | No | ACR 50; PsARC; Function (HAQ-DI continuous); Function (HAQ-DI dichotomous); Disease activity (DAPSA, DAS28, CDAI or MDA) as continuous outcome; Disease activity (DAPSA, DAS28, CDAI or MDA) as dichotomous outcome; Pain; Quality of life; Skin disease (PASI dichotomous); Serious adverse events; Total adverse events |
|  | 132 | Tofacitinib | Oral | 10 | Twice daily |  |  |
|  | 131 | Placebo | Oral | NA | Twice daily |  |  |
| Kavanaugh 2009 | 146 | Golimumab | SC | 50 | At weeks 0, 4, 8, 12, 16, and 20 | No | ACR 50; PsARC; Function (HAQ-DI continuous); Disease activity (DAPSA, DAS28, CDAI or MDA) as continuous outcome; Quality of life; Skin disease (PASI dichotomous) |
|  | 146 | Golimumab | SC | 100 | At weeks 0, 4, 8, 12, 16, and 20 |  |  |
|  | 113 | Placebo | SC | NA | At weeks 0, 4, 8, 12, 16, and 20 |  |  |
| Kavanaugh 2017 | 241 | Golimumab | IV | 2 mg/kg | At weeks 0 and 4 and every 8 weeks | No | ACR 50; Function (HAQ-DI continuous); Disease activity (DAPSA, DAS28, CDAI or MDA) as continuous outcome; Disease activity (DAPSA, DAS28, CDAI or MDA) as dichotomous outcome; Quality of life; Skin disease (PASI continuous); Skin disease (PASI dichotomous) |
|  | 239 | Placebo | IV | NA | At weeks 0 and 4 and every 8 weeks |  |  |
| Kivitz 2019 | 114 | Secukinumab (LD) | SC | 150 | At weeks 0, 1, 2and 3 followed by dosing every 4 weeks starting at week 4 | No | ACR 50; Function (HAQ-DI dichotomous); Disease activity (DAPSA, DAS28, CDAI or MDA) as continuous outcome; Disease activity (DAPSA, DAS28, CDAI or MDA) as dichotomous outcome; Quality of life; Skin disease (PASI dichotomous) |
|  | 113 | Secukinumab (without) | SC | 150 | At weeks 0, 1, 2and 3 followed by dosing every 4 weeks starting at week 4 |  |  |
|  | 114 | Placebo | SC | NA | At weeks 0, 1, 2and 3 followed by dosing every 4 weeks starting at week 4 |  |  |
| McInnes 2014 | 28 | Secukinumab | IV | 10 mg/kg | At baseline (day 1) and on day 22. | No | ACR 50; PsARC; Function (HAQ-DI continuous); Function (HAQ-DI dichotomous); Disease activity (DAPSA, DAS28, CDAI or MDA) as continuous outcome; Disease activity (DAPSA, DAS28, CDAI or MDA) as dichotomous outcome; Quality of life; Skin disease (PASI continuous); Serious adverse events; Total adverse events |
|  | 14 | Placebo | IV | NA | At baseline (day 1) and on day 22. |  |  |
| McInnes 2015 | 99 | Secukinumab | SC | 75 | Once a week from baseline to week 4 and then every 4 weeks thereafter | No | ACR 50; Function (HAQ-DI continuous); Disease activity (DAPSA, DAS28, CDAI or MDA) as continuous outcome; Quality of life; Skin disease (PASI dichotomous); Serious adverse events; Total adverse events |
|  | 100 | Secukinumab | SC | 150 | Once a week from baseline to week 4 and then every 4 weeks thereafter |  |  |
|  | 100 | Secukinumab | SC | 300 | Once a week from baseline to week 4 and then every 4 weeks thereafter |  |  |
|  | 98 | Placebo | SC | NA | Once a week from baseline to week 4 and then every 4 weeks thereafter |  |  |
| McInnes 2020 | 427 | Adalimumab | SC | 40 | Every 2 weeks from baseline until week 50 | No | ACR 50; Function (HAQ-DI continuous); Function (HAQ-DI dichotomous); Disease activity (DAPSA, DAS28, CDAI or MDA) as dichotomous outcome; Skin disease (PASI dichotomous); Serious adverse events; Total adverse events |
|  | 426 | Secukinumab | SC | 300 | At baseline, weeks 1, 2, 3, and 4, and then every 4 weeks until week 48 |  |  |
| McInnes 2021 | 429 | Adalimumab | SC | 40 | Every other week | No | ACR 50; Function (HAQ-DI continuous); Disease activity (DAPSA, DAS28, CDAI or MDA) as dichotomous outcome; Pain; Quality of life; Skin disease (PASI dichotomous); Serious adverse events; Total adverse events |
|  | 423 | Placebo | Oral | NA | Once daily |  |  |
| Mease 2000 | 30 | Etanercept | SC | 25 | Twice weekly by subcutaneous administration for 12 weeks | No | ACR 50; PsARC; Function (HAQ-DI continuous); Skin disease (PASI dichotomous); Serious adverse events |
|  | 30 | Placebo | SC | NA | Twice weekly by subcutaneous administration for 12 weeks |  |  |
| Mease 2004 | 101 | Etanercept | SC | 25 | Twice weekly | No | ACR 50; PsARC; Function (HAQ-DI continuous); Quality of life; Skin disease (PASI dichotomous); Serious adverse events |
|  | 104 | Placebo | SC | NA | Twice weekly |  |  |
| Mease 2005 | 153 | Adalimumab | SC | 40 | Given every other week | No | ACR 50; PsARC; Function (HAQ-DI continuous); Quality of life; Skin disease (PASI dichotomous); Serious adverse events |
|  | 162 | Placebo | SC | NA | Given every other week |  |  |
| Mease 2014 | 138 | Certolizumab pegol | SC | 200 | Every 2 weeks | No | ACR 50; PsARC; Function (HAQ-DI continuous); Disease activity (DAPSA, DAS28, CDAI or MDA) as dichotomous outcome; Pain; Quality of life; Skin disease (PASI dichotomous); Serious adverse events; Total adverse events |
|  | 135 | Certolizumab pegol | SC | 400 | Every 4 weeks |  |  |
|  | 136 | Placebo | SC | NA | Nr |  |  |
| Mease 2015 | 202 | Secukinumab | SC | 75 | At baseline and weeks 2 and 4, followed by subcutaneous secukinumab at a dose of either 150 mg or 75 mg every 4 weeks thereafter. | No | ACR 50; Function (HAQ-DI continuous); Disease activity (DAPSA, DAS28, CDAI or MDA) as continuous outcome; Pain; Quality of life; Skin disease (PASI dichotomous); Serious adverse events; Total adverse events |
|  | 202 | Secukinumab | SC | 150 | At baseline and weeks 2 and 4, followed by subcutaneous secukinumab at a dose of either 150 mg or 75 mg every 4 weeks thereafter. |  |  |
|  | 202 | Placebo | SC | NA | At baseline and weeks 2 and 4, followed by subcutaneous secukinumab at a dose of either 150 mg or 75 mg every 4 weeks thereafter. |  |  |
| Mease 2017a | 101 | Adalimumab | SC | 40 | Every 2 weeks | No | ACR 50; Function (HAQ-DI continuous); Function (HAQ-DI dichotomous); Disease activity (DAPSA, DAS28, CDAI or MDA) as continuous outcome; Pain; Quality of life; Skin disease (PASI dichotomous); Serious adverse events; Total adverse events |
|  | 106 | Placebo | SC | NA | Every 2 weeks |  |  |
| Mease 2017b | 106 | Adalimumab | SC | 40 | Once every 2 weeks | No | ACR 50; PsARC; Function (HAQ-DI continuous); Function (HAQ-DI dichotomous); Disease activity (DAPSA, DAS28, CDAI or MDA) as continuous outcome; Disease activity (DAPSA, DAS28, CDAI or MDA) as dichotomous outcome; Pain; Quality of life; Skin disease (PASI dichotomous); Serious adverse events; Total adverse events |
|  | 107 | Tofacitinib | Oral | 5 | Twice daily |  |  |
|  | 104 | Tofacitinib | Oral | 10 | Twice daily |  |  |
|  | 105 | Placebo | Oral | NA | Twice daily |  |  |
| Mease 2018 | 220 | Secukinumab | SC | 150 | Every week | No | ACR 50; Function (HAQ-DI continuous); Disease activity (DAPSA, DAS28, CDAI or MDA) as continuous outcome; Disease activity (DAPSA, DAS28, CDAI or MDA) as dichotomous outcome; Skin disease (PASI dichotomous); Serious adverse events; Total adverse events |
|  | 222 | Secukinumab (LD) | SC | 150 | Every week |  |  |
|  | 222 | Secukinumab | SC | 300 | Every week |  |  |
|  | 332 | Placebo | SC | NA | Every week |  |  |
| Mease 2019 | 284 | Etanercept | SC | 50 | Weekly | No | ACR 50; Function (HAQ-DI continuous); Disease activity (DAPSA, DAS28, CDAI or MDA) as continuous outcome; Disease activity (DAPSA, DAS28, CDAI or MDA) as dichotomous outcome; Quality of life |
|  | 283 | Etanercept + MTX | SC + Oral | 50 + 20 | Weekly |  |  |
|  | 284 | MTX | Oral | 20 | Weekly |  |  |
| Nash 2018 | 138 | Secukinumab | SC | 150 | At baseline, weeks 1, 2, 3, and 4, and every 4 weeks thereafter | No | ACR 50; Function (HAQ-DI continuous); Disease activity (DAPSA, DAS28, CDAI or MDA) as continuous outcome; Pain; Quality of life; Skin disease (PASI dichotomous); Serious adverse events; Total adverse events |
|  | 139 | Secukinumab | SC | 300 | At baseline, weeks 1, 2, 3, and 4, and every 4 weeks thereafter |  |  |
|  | 137 | Placebo | SC | NA | At baseline, weeks 1, 2, 3, and 4, and every 4 weeks thereafter |  |  |
| Nguyen 2019 | 103 | Secukinumab | SC | 150 | Every 4 weeks (q4w) to week 16 | No | ACR 50; Function (HAQ-DI continuous); Disease activity (DAPSA, DAS28, CDAI or MDA) as continuous outcome; Disease activity (DAPSA, DAS28, CDAI or MDA) as dichotomous outcome; Quality of life; Skin disease (PASI dichotomous); Serious adverse events; Total adverse events |
|  | 103 | Secukinumab | SC | 300 | Every 4 weeks (q4w) to week 16 |  |  |
|  | 102 | Placebo | SC | NA | Every 4 weeks (q4w) to week 16 |  |  |
| van Kuijk 2009 | 12 | Adalimumab | SC | 40 | Every other week | No | Disease activity (DAPSA, DAS28, CDAI or MDA) as continuous outcome; Skin disease (PASI continuous) |
|  | 12 | Placebo | SC | NA | Every other week |  |  |
| vanMens 2019 | 26 | Golimumab | SC | 50 | Monthly | No | ACR 50; Function (HAQ-DI continuous); Disease activity (DAPSA, DAS28, CDAI or MDA) as continuous outcome; Disease activity (DAPSA, DAS28, CDAI or MDA) as dichotomous outcome; Pain; Quality of life; Skin disease (PASI continuous); Serious adverse events; Total adverse events |
|  | 25 | Placebo | SC | NA | Monthly |  |  |
| Vieira-Sousa 2020 | 21 | Golimumab | SC | 50 | Every 4 weeks for 24 weeks | Yes | ACR 50; PsARC; Function (HAQ-DI continuous); Disease activity (DAPSA, DAS28, CDAI or MDA) as continuous outcome; Disease activity (DAPSA, DAS28, CDAI or MDA) as dichotomous outcome; Skin disease (PASI continuous); Skin disease (PASI dichotomous); Serious adverse events; Total adverse events |
|  | 23 | Placebo | SC | NA | Every 4 weeks for 24 weeks |  |  |

Abbreviations: IV, intravenous; NA, not applicable; NR; not reported; SC, subcutaneous

Results of Primary outcomes

Disease response (ACR 50)

Figure S1. Network meta-analysis estimates of interventions versus placebo for disease response outcome (ACR 50) at 6-month follow-up

Abbreviations: ADA, adalimumab; CER, certolizumab pegol; ETA, etanercept; GOL, golimumab; IFN, infliximab; SEC, secukinumab; PBO, placebo

Table S6. League table of relative effects of interventions for disease response outcome (ACR 50) at 6-month follow up

| **ADA** | . | . | . | . | 3.73 (2.46, 5.65) | 0.93 (0.41, 2.10) | 0.82 (0.34, 1.96) |
| --- | --- | --- | --- | --- | --- | --- | --- |
| 1.06 (0.39, 2.88) | **CER** | . | . | . | 3.37 (1.34, 8.49) | . | . |
| 1.32 (0.63, 2.79) | 1.24 (0.40, 3.84) | **ETA** | . | . | 2.71 (1.42, 5.18) | . | . |
| 1.14 (0.55, 2.36) | 1.07 (0.35, 3.27) | 0.86 (0.35, 2.12) | **GOL** | . | 3.15 (1.68, 5.90) | . | . |
| 0.94 (0.43, 2.10) | 0.89 (0.28, 2.84) | 0.72 (0.27, 1.86) | 0.83 (0.32, 2.14) | **IFX** | 3.79 (1.87, 7.68) | . | . |
| 3.58 (2.47, 5.19) | 3.37 (1.34, 8.49) | 2.71 (1.42, 5.18) | 3.15 (1.68, 5.90) | 3.79 (1.87, 7.68) | **PBO** | 0.24 (0.16, 0.35) | 0.36 (0.18, 0.72) |
| 0.87 (0.54, 1.38) | 0.82 (0.30, 2.20) | 0.66 (0.31, 1.38) | 0.76 (0.37, 1.58) | 0.92 (0.42, 2.03) | 0.24 (0.17, 0.35) | **SEC** | . |
| 1.15 (0.60, 2.20) | 1.09 (0.36, 3.30) | 0.87 (0.36, 2.14) | 1.01 (0.42, 2.45) | 1.22 (0.48, 3.12) | 0.32 (0.17, 0.60) | 1.33 (0.66, 2.69) | TOF |

Each cell in the table above contains the relative risk (RR) and 95% confidence interval of the intervention in the respective column versus the comparator in the respective row. The Lower triangle presents the result of the network meta-analysis and the upper triangle presents the result of the pairwise meta-analysis. RRs greater than 1 favor treatment on the left.

Abbreviations: ADA, adalimumab; CER, certolizumab pegol; ETA, etanercept; GOL, golimumab; IFN, infliximab; SEC, secukinumab; PBO, placebo; TOF, tofacitinib

Figure S2. Ranking (P-score) of interventions for disease response outcome (ACR 50) at 6-month follow-up

Abbreviations: ADA, adalimumab; CER, certolizumab pegol; ETA, etanercept; GOL, golimumab; IFN, infliximab; SEC, secukinumab; PBO, placebo

Disease response (PsARC)

Figure S3. Network meta-analysis estimates of interventions versus placebo for disease response outcome (PsARC) at 6-month follow-up

Abbreviations: ADA, adalimumab; CER, certolizumab pegol; ETA, etanercept; GOL, golimumab; IFN, infliximab; PBO, placebo; TOF, tofacitinib

Table S7. League table of relative effects of interventions for disease response outcome (PsARC) at 6-month follow up

| **ADA** | . | . | . | . | 1.88 (1.35, 2.61) | 1.19 (0.68, 2.10) |
| --- | --- | --- | --- | --- | --- | --- |
| 0.83 (0.43, 1.59) | **CER** | . | . | . | 2.35 (1.33, 4.14) | . |
| 0.59 (0.33, 1.08) | 0.72 (0.34, 1.53) | **ETA** | . | . | 3.27 (1.98, 5.42) | . |
| 1.67 (0.84, 3.29) | 2.01 (0.88, 4.59) | 2.80 (1.28, 6.14) | **GOL** | . | 1.17 (0.64, 2.13) | . |
| 0.74 (0.42, 1.30) | 0.89 (0.43, 1.85) | 1.24 (0.62, 2.47) | 0.44 (0.21,0.95) | **INF** | 2.64 (1.65, 4.22) | . |
| 1.95 (1.41, 2.69) | 2.35 (1.33, 4.14) | 3.27 (1.98, 5.42) | 1.17 (0.64, 2.13) | 2.64 (1.65, 4.22) | **PBO** | 0.66 (0.43, 1.00) |
| 1.20 (0.77, 1.87) | 1.45 (0.73, 2.89) | 2.02 (1.07, 3.84) | 0.72 (0.35, 1.48) | 1.63 (0.88, 3.01) | 0.62 (0.42, 0.92) | **TOF** |

Each cell in the table above contains the relative risk (RR) and 95% confidence interval of the intervention in the respective column versus the comparator in the respective row. The Lower triangle presents the result of the network meta-analysis and the upper triangle presents the result of the pairwise meta-analysis. RRs greater than 1 favor treatment on the left.

Abbreviations: ADA, adalimumab; CER, certolizumab pegol; ETA, etanercept; GOL, golimumab; IFN, infliximab; PBO, placebo; TOF, tofacitinib

Figure S4. Ranking (P-score) of interventions for disease response outcome (PsARC) at 6-month follow-up

Abbreviations: ADA, adalimumab; CER, certolizumab pegol; ETA, etanercept; GOL, golimumab; IFN, infliximab; PBO, placebo; TOF, tofacitinib

Serious adverse events

Figure S5. Network meta-analysis estimates of interventions versus placebo for serious adverse events outcome

Abbreviations: ADA, adalimumab; CER, certolizumab pegol; ETA, etanercept; GOL, golimumab; IFN, infliximab; SEC, secukinumab; PBO, placebo; TOF, tofacitinib

Table S8. League table of relative effects of interventions for serious adverse events outcome

| **ADA** | . | . | . | . | 1.18 (0.69,2.01) | 0.85 (0.52,1.38) | 0.34 (0.04,3.18) |
| --- | --- | --- | --- | --- | --- | --- | --- |
| 0.51 (0.19, 1.35) | **CER** | . | . | . | 1.74 (0.72,4.22) | . | . |
| 1.03 (0.28, 3.82) | 2.02 (0.44, 9.32) | **ETA** | . | . | 0.86 (0.25,3.01) | . | . |
| 2.59 (0.27,24.98) | 5.10 (0.46,56.02) | 2.53 (0.20,32.46) | **GOL** | . | 0.34 (0.04,3.17) | . | . |
| 0.44 (0.06, 3.51) | 0.87 (0.10, 7.96) | 0.43 (0.04, 4.67) | 0.17 (0.01, 3.47) | **IFX** | 2.00 (0.26 a15.24) | . | . |
| 0.89 (0.59, 1.34) | 1.74 (0.72, 4.22) | 0.86 (0.25, 3.01) | 0.34 (0.04, 3.17) | 2.00 (0.26,15.24) | **PBO** | 1.37 (0.90,2.09) | 1.01 (0.21,4.94) |
| 1.04 (0.70, 1.54) | 2.04 (0.79, 5.31) | 1.01 (0.28, 3.71) | 0.40 (0.04, 3.83) | 2.35 (0.30,18.44) | 1.17 (0.81, 1.69) | **SEC** | . |
| 0.72 (0.17, 2.98) | 1.41 (0.27, 7.39) | 0.70 (0.11, 4.56) | 0.28 (0.02, 3.84) | 1.62 (0.14,19.08) | 0.81 (0.20, 3.28) | 0.69 (0.17, 2.89) | **TOF** |

Each cell in the table above contains the relative risk (RR) and 95% confidence interval of the intervention in the respective column versus the comparator in the respective row. The Lower triangle presents the result of the network meta-analysis and the upper triangle presents the result of the pairwise meta-analysis. RRs greater than 1 favor treatment on the left.

Abbreviations: ADA, adalimumab; CER, certolizumab pegol; ETA, etanercept; GOL, golimumab; IFN, infliximab; SEC, secukinumab; PBO, placebo; TOF, tofacitinib

Figure S6. Ranking (P-score) of interventions for serious adverse events outcome

Abbreviations: ADA, adalimumab; CER, certolizumab pegol; ETA, etanercept; GOL, golimumab; IFN, infliximab; SEC, secukinumab; PBO, placebo; TOF, tofacitinib

Results of secondary outcomes

|  |  |  |
| --- | --- | --- |
| (a) HAQ-DI dichotomous | (b) HAQ-DI continuous | (c) pain |
|  |  |  |
| (d) DAS 28 dichotomous | (e) DAS 28 continuous | (f) SF-36 PCS |
|  |  |  |
| (g) PASI dichotomous | (h) PASI continuous | (i) Total adverse events |

Figure S7. Network diagram of the secondary outcomes evaluated in included randomized clinical trials

Abbreviations: ADA, adalimumab; CER, certolizumab pegol; ETA, etanercept; IFN, infliximab; SEC, secukinumab; PBO, placebo; TOF, tofacitinib

Function (HAQ-DI)

Figure S8. Network meta-analysis estimates of interventions versus placebo for function outcome (HAQ-DI)

Abbreviations: ADA, adalimumab; IFN, infliximab; SEC, secukinumab; TOF, tofacitinib

Table S9. League table of relative effects of interventions for function outcome (HAQ-DI)

| (a) dichotomous | | | | |  | (b) continuous | | | |
| --- | --- | --- | --- | --- | --- | --- | --- | --- | --- |
| **ADA** | . | 1.48 (1.22,1.80) | . | 1.00 (0.75,1.33) |  | **ADA** | 0.03 (-0.24,0.30) | 0.03 (-0.26,0.32) | 0.02 (-0.06,0.10) |
| 0.58 (0.36,0.94) | **IFX** | 2.60 (1.66,4.07) | . | . |  | 0.03 (-0.24,0.30) | **ETA** | 0.00 (-0.39,0.39) | . |
| 1.51 (1.25,1.82) | 2.60 (1.66,4.07) | **PBO** | 0.68 (0.47,0.99) | 0.57 (0.44,0.73) |  | 0.03 (-0.26,0.32) | 0.00 (-0.39,0.39) | **INF** | . |
| 1.03 (0.68,1.56) | 1.77 (0.99,3.18) | 0.68 (0.47,0.99) | **SEC** | . |  | 0.02 (-0.06,0.10) | -0.01 (-0.29,0.27) | -0.01 (-0.31,0.29) | **SEC** |
| 0.91 (0.72,1.16) | 1.58 (0.95,2.61) | 0.61 (0.48,0.76) | 0.89 (0.57,1.38) | **TOF** |  |  |  |  |  |

Each cell in the above table contains the relative risk (RR) or mean difference (MD) and 95% confidence interval of the intervention in the respective column versus the comparator in the respective row. The Lower triangle presents the result of the network meta-analysis and the upper triangle presents the result of the pairwise meta-analysis. RRs greater than 1 favor treatment on the left and MDs less than zero favor treatment on the left.

Abbreviations: ADA, adalimumab; ETA, etanercept; IFN, infliximab; SEC, secukinumab; PBO, placebo; TOF, tofacitinib

|  |  |
| --- | --- |
| (a) dichotomous | (b) continuous |

Figure S9. Ranking of interventions for function outcome (HAQ-DI)

Abbreviations: ADA, adalimumab; ETA, etanercept; IFN, infliximab; SEC, secukinumab; PBO, placebo; TOF, tofacitinib

Table S10. Direct and indirect comparisons between interventions for function outcome measured by Health Assessment Questionnaire Disability Index (HAQ-DI)

| **Measurement method** | **Comparison** | **Follow-up** | **N. of studies** | **Effect measure** | **Direct estimate (95% CI)** | **Indirect estimate (95% CI)** |
| --- | --- | --- | --- | --- | --- | --- |
| *Dichotomous results* | | | | | | |
| HAQ-DI | ADA x SEC | 12 | 1 | RR | 0.93 (0.82 to 1.05) |  |
| *Continuous results* | | | | | | |
| HAQ-DI | ADA x ETA | 12 | 1 | MD | 0.03 (-0.24 to 0.30) |  |
| HAQ-DI | ADA x IFN | 12 | 1 | MD | 0.03 (-0.26 to 0.32) |  |
| HAQ-DI | ETA x IFN | 12 | 1 | MD | 0.00 (-0.39 to 0.39) |  |
| HAQ-DI | ADA x SEC | 12 | 1 | MD | 0.02 (-0.06 to 0.10) |  |
| HAQ-DI | ETA x SEC | 12 | - | MD |  | -0.01 (-0.29 to 0.27) |
| HAQ-DI | IFN x SEC | 12 | - | MD |  | -0.01 (-0.31 to 0.29) |

Abbreviations: ADA, adalimumab; CI, confidence interval; ETA, etanercept; HAQ-DI, Health Assessment Questionnaire Disability Index; IFN, infliximab; MD, mean difference; RR, risk ratio; SEC, Secukinumab.

Disease activity

|  |  |
| --- | --- |
| (a) dichotomous | (b) continuous |

Figure S10. Network meta-analysis estimates the effect of interventions compared for placebo on disease activity outcome (DAS-28)

Abbreviations: ADA, adalimumab; CER, certolizumab pegol; CI, confidence interval; ETA, etanercept; GOL, golimumab; IFN, infliximab; SEC, secukinumab; SMD, standardized mean difference; PBO, placebo; RR, risk ratio; TOF, tofacitinib

Table S11. League table of relative effects of interventions on disease activity outcome (DAS-28)

| (a) dichotomous | | | | | | | |
| --- | --- | --- | --- | --- | --- | --- | --- |
| **ADA** | . | . | . | . | 2.95 (2.03, 4.30) | . | 0.97 (0.53, 1.80) |
| 0.47 (0.20, 1.14) | **CER** | . | . | . | 5.73 (2.56,12.83) | . | . |
| 1.73 (0.94, 3.18) | 3.67 (1.43, 9.45) | **ETA** | . | . | 1.56 (0.95, 2.55) | . | . |
| 1.62 (0.92, 2.87) | 3.44 (1.37, 8.64) | 0.94 (0.48, 1.82) | **GOL** | . | 1.67 (1.07, 2.60) | . | . |
| 0.96 (0.54, 1.69) | 2.03 (0.81, 5.09) | 0.55 (0.29, 1.07) | 0.59 (0.32, 1.10) | **INF** | 2.82 (1.82, 4.38) | . | . |
| 2.70 (1.89, 3.86) | 5.73 (2.56,12.83) | 1.56 (0.95, 2.55) | 1.67 (1.07, 2.60) | 2.82 (1.82, 4.38) | **PBO** | 0.28 (0.17, 0.45) | 0.46 (0.27, 0.78) |
| 0.75 (0.41, 1.37) | 1.58 (0.62, 4.07) | 0.43 (0.22, 0.86) | 0.46 (0.24, 0.89) | 0.78 (0.40, 1.51) | 0.28 (0.17, 0.45) | **SEC** | . |
| 1.18 (0.73, 1.91) | 2.51 (0.99, 6.35) | 0.68 (0.35, 1.34) | 0.73 (0.38, 1.38) | 1.23 (0.65, 2.34) | 0.44 (0.28, 0.69) | 1.58 (0.81, 3.10) | **TOF** |

| (b) continuous | | | | | | |
| --- | --- | --- | --- | --- | --- | --- |
| **ADA** | . | . | . | -1.00 (-1.45, -0.54) | . | -0.19 (-1.10, 0.71) |
| -1.04 (-2.03,-0.04) | **ETA** | . | . | 0.03 (-0.85, 0.92) | . | . |
| -0.07 (-0.80, 0.66) | 0.96 (-0.09, 2.02) | **GOL** | . | -0.93 (-1.51, -0.36) | . | . |
| 2.31 (1.39, 3.22) | 3.34 (2.15, 4.54) | 2.38 (1.39, 3.37) | **INF** | -3.31 (-4.11, -2.51) | . | . |
| -1.00 (-1.45,-0.56) | 0.03 (-0.85, 0.92) | -0.93 (-1.51,-0.36) | -3.31 (-4.11,-2.51) | **PBO** | 0.63 (0.26, 1.00) | 0.61 (-0.03, 1.25) |
| -0.37 (-0.95, 0.20) | 0.66 (-0.30, 1.62) | -0.30 (-0.98, 0.38) | -2.68 (-3.56,-1.80) | 0.63 (0.26, 1.00) | **SEC** | . |
| -0.32 (-0.99, 0.35) | 0.72 (-0.36, 1.79) | -0.25 (-1.09, 0.59) | -2.63 (-3.63,-1.62) | 0.68 (0.08, 1.29) | 0.05 (-0.66, 0.76) | **TOF** |

Each cell in the above table contains the risk ratio (RR) or standardized mean difference (SMD) and 95% confidence interval of the intervention in the respective column versus the comparator in the respective row. The Lower triangle presents the result of the network meta-analysis and the upper triangle presents the result of the pairwise meta-analysis. RR greater than 1 favor treatment on the left and SMD less than zero favor treatment on the left.

Abbreviations: ADA, adalimumab; CER, certolizumab pegol; ETA, etanercept; GOL, golimumab; IFN, infliximab; SEC, secukinumab; PBO, placebo; TOF, tofacitinib

|  |  |
| --- | --- |
| (a) dichotomous | (b) continuous |

Figure S11. Ranking of interventions based on their effect on disease activity outcome (DAS-28)

Abbreviations: ADA, adalimumab; CER, certolizumab pegol; ETA, etanercept; GOL, golimumab; IFN, infliximab; PBO, placebo; SEC, secukinumab; TOF, tofacitinib

Pain

Figure S12. Network meta-analysis estimates the effect of interventions compared to placebo on pain outcome

Abbreviations: ADA, adalimumab; CER, certolizumab pegol; CI, confidence interval; GOL, golimumab; IFN, infliximab; MD, mean difference; SEC, secukinumab; PBO, placebo; TOF, tofacitinib

Table S12. League table of relative effects of interventions on pain outcome (0 to100 scale)

| **ADA** | . | . | . | -11.20 (-23.92, 1.53) | . | -0.38 (-25.84,25.08) |
| --- | --- | --- | --- | --- | --- | --- |
| 5.90 (-22.12,33.93) | **CER** | . | . | -17.30 (-42.38, 7.78) | . | . |
| 12.61 (-19.85,45.07) | 6.71 (-32.36,45.78) | **GOL** | . | -24.01 (-53.97, 5.95) | . | . |
| 8.29 (-11.03,27.61) | 2.39 (-26.70,31.48) | -4.32 (-37.70,29.06) | **INF** | -19.69 (-34.42,-4.96) | . | . |
| -11.40 (-23.90, 1.10) | -17.30 (-42.38, 7.78) | -24.01 (-53.97, 5.95) | -19.69 (-34.42,-4.96) | **PBO** | 8.65 (-3.88,21.17) | 12.61 (-5.36,30.58) |
| -2.75 (-20.44,14.95) | -8.65 (-36.69,19.38) | -15.36 (-47.83,17.11) | -11.04 (-30.37, 8.30) | 8.65 (-3.88,21.17) | **SEC** | . |
| 0.95 (-17.93,19.83) | -4.95 (-35.26,25.35) | -11.66 (-46.11,22.79) | -7.34 (-29.84,15.16) | 12.35 (-4.66,29.36) | 3.70 (-17.42,24.82) | **TOF** |

Each cell in the table above contains the mean difference (MD) and 95% confidence interval of the intervention in the respective column versus the comparator in the respective row. The Lower triangle presents the result of the network meta-analysis and the upper triangle presents the result of the pairwise meta-analysis. MD smaller than zero favor treatment on the left.

Abbreviations: ADA, adalimumab; CER, certolizumab pegol; GOL, golimumab; IFN, infliximab; SEC, secukinumab; PBO, placebo; TOF, tofacitinib

Figure S13. Ranking of interventions based on their effect on pain outcome

Abbreviations: ADA, adalimumab; CER, certolizumab pegol; GOL, golimumab; IFN, infliximab; SEC, secukinumab; PBO, placebo; TOF, tofacitinib

Quality of life

Figure S14. Network meta-analysis estimates the effect of interventions compared to placebo on quality of life outcome, as measured by the Form 36 - physical component summary (SF-36 PCS)

Abbreviations: ADA, adalimumab; CER, certolizumab pegol; CI, confidence interval; ETA, etanercept; GOL, golimumab; IFN, infliximab; MD, mean difference; SEC, secukinumab; PBO, placebo; TOF, tofacitinib

Table S13. League table of relative effects of interventions on quality of life outcome

| **ADA** | . | . | . | . | 4.15 (2.58, 5.73) | . | 0.00 (-4.08, 4.08) |
| --- | --- | --- | --- | --- | --- | --- | --- |
| -1.76 (-5.78, 2.25) | **CER** | . | . | . | 5.90 (2.20, 9.60) | . | . |
| -0.64 (-3.88, 2.60) | 1.12 (-3.55, 5.79) | **ETA** | . | . | 4.78 (1.93, 7.62) | . | . |
| 0.32 (-3.13, 3.76) | 2.08 (-2.73, 6.89) | 0.96 (-3.23, 5.14) | **GOL** | . | 3.82 (0.75, 6.89) | . | . |
| -2.26 (-6.71, 2.18) | -0.50 (-6.07, 5.07) | -1.62 (-6.67, 3.42) | -2.58 (-7.75, 2.59) | **INF** | 6.40 (2.24,10.56) | . | . |
| 4.14 (2.58, 5.69) | 5.90 (2.20, 9.60) | 4.78 (1.93, 7.62) | 3.82 (0.75, 6.89) | 6.40 (2.24,10.56) | **PBO** | -3.74 (-5.37,-2.10) | -3.21 (-5.91,-0.51) |
| 0.40 (-1.86, 2.65) | 2.16 (-1.88, 6.21) | 1.04 (-2.24, 4.32) | 0.08 (-3.39, 3.56) | 2.66 (-1.81, 7.14) | -3.74 (-5.37,-2.10) | **SEC** | . |
| 0.67 (-2.09, 3.44) | 2.44 (-2.06, 6.93) | 1.31 (-2.51, 5.13) | 0.36 (-3.63, 4.34) | 2.94 (-1.94, 7.82) | -3.46 (-6.01,-0.92) | 0.27 (-2.75, 3.30) | **TOF** |

Each cell in table above contains the mean difference (MD) and 95% confidence interval of the intervention in the respective column versus the comparator in the respective row. The Lower triangle presents the result of the network meta-analysis and the upper triangle presents the result of the pairwise meta-analysis. MD greater than zero favor treatment on the left.

Abbreviations: ADA, adalimumab; CER, certolizumab pegol; ETA, etanercept; GOL, golimumab; IFN, infliximab; SEC, secukinumab; PBO, placebo; TOF, tofacitinib

Figure S15. Ranking of interventions and placebo based on their effect on quality of life outcome, as measured by Form 36 - physical component summary (SF-36 PCS)

Abbreviations: ADA, adalimumab; CER, certolizumab pegol; ETA, etanercept; GOL, golimumab; IFN, infliximab; SEC, secukinumab; PBO, placebo; TOF, tofacitinib

Skin disease

|  |  |
| --- | --- |
| (a) dichotomous | (b) continuous |

Figure S16. Network meta-analysis estimates the effect of interventions compared to placebo on skin disease outcome, as measured by Psoriasis Area and Severity Index (PASI)

Abbreviations: ADA, adalimumab; CER, certolizumab pegol; CI, confidence interval; MD, mean difference; ETA, etanercept; GOL, golimumab; IFN, infliximab; SEC, secukinumab; PBO, placebo; RR, risk ratio; TOF, tofacitinib

Table S14. League table of relative effects of interventions on skin disease outcome

| (a) dichotomous | | | | | | | |  | (b) continuous | | | |
| --- | --- | --- | --- | --- | --- | --- | --- | --- | --- | --- | --- | --- |
| **ADA** | . | . | . | . | 3.17 (1.94, 5.17) | . | 0.91 (0.34, 2.42) |  | **ADA** | . | . | -1.22 (-3.09, 0.66) |
| 0.74 (0.24, 2.33) | **CER** | . | . | . | 4.06 (1.44,11.47) | . | . |  | -0.72 (-2.89, 1.44) | **GOL** | . | -0.49 (-1.57, 0.58) |
| 0.38 (0.08, 1.80) | 0.52 (0.09, 3.13) | **ETA** | . | . | 7.86 (1.81,34.21) | . | . |  | 6.48 (4.42, 8.54) | 7.21 (5.83, 8.58) | **INF** | -7.70 (-8.55,-6.85) |
| 1.13 (0.38, 3.33) | 1.53 (0.37, 6.31) | 2.95 (0.51,17.16) | **GOL** | . | 2.66 (1.01, 7.01) | . | . |  | -1.22 (-3.09, 0.66) | -0.49 (-1.57, 0.58) | -7.70 (-8.55, -6.85) | **PBO** |
| 0.82 (0.31, 2.14) | 1.10 (0.29, 4.18) | 2.13 (0.39,11.57) | 0.72 (0.20, 2.59) | **INF** | 3.68 (1.60, 8.48) | . | . |  |  |  |  |  |
| 3.01 (1.87, 4.87) | 4.06 (1.44,11.47) | 7.86 (1.81,34.21) | 2.66 (1.01, 7.01) | 3.68 (1.60, 8.48) | **PBO** | 0.20 (0.13, 0.32) | 0.47 (0.22, 1.01) |  |  |  |  |  |
| 0.62 (0.32, 1.20) | 0.83 (0.27, 2.58) | 1.61 (0.34, 7.50) | 0.54 (0.19, 1.59) | 0.75 (0.29, 1.95) | 0.20 (0.13, 0.32) | **SEC** | . |  |  |  |  |  |
| 1.26 (0.59, 2.66) | 1.70 (0.48, 5.96) | 3.28 (0.64,16.78) | 1.11 (0.34, 3.69) | 1.54 (0.51, 4.59) | 0.42 (0.21, 0.85) | 2.04 (0.88, 4.76) | **TOF** |  |  |  |  |  |

Each cell of the table above contains the risk ratio (RR) or mean difference (MD) and 95% confidence interval of the intervention in the respective column versus the comparator in the respective row. The Lower triangle presents the result of the network meta-analysis and the upper triangle presents the result of the pairwise meta-analysis. RR greater than 1 favor treatment on the left and MD less than zero favor treatment on the left.

Abbreviations: ADA, adalimumab; CER, certolizumab pegol; ETA, etanercept; GOL, golimumab; IFN, infliximab; SEC, secukinumab; PBO, placebo; TOF, tofacitinib

|  |  |
| --- | --- |
| (a) dichotomous | (b) continuous |

Figure S17. Ranking of interventions based on their effect on skin disease outcome, as measured by Psoriasis Area and Severity Index (PASI)

Abbreviations: ADA, adalimumab; CER, certolizumab pegol; ETA, etanercept; GOL, golimumab; IFN, infliximab; SEC, secukinumab; TOF, tofacitinib

Table S15. Direct and indirect comparisons between interventions for Disease Activity outcome, as measured by Psoriasis Area and Severity Index (PASI)

| **Measured method** |  | **Comparison** | **Follow-up** | **N. of studies** | **Effect measured** | **Direct estimates (95% CI)** | |
| --- | --- | --- | --- | --- | --- | --- | --- |
| *Dichotomous results* | | | | | | |  |
| PASI 75 |  | ADA x SEC | 12 | 1 | RR | 0.77 (0.68 to 0,88) | |
| *Continuous results* | | | | | | |  |
| PASI |  | ADA x ETA | 12 | 1 | MD | -1.46 (-2.67 to -0.25) | |
| PASI |  | ADA x IFN | 12 | 1 | MD | 0.34 (-0.23 to 0.91) | |
| PASI |  | ETA x IFN | 12 | 1 | MD | 1.80 (0.66 to 2.94) | |

Abbreviations: ADA, adalimumab; CI, confidence interval; ETA, etanercept; IFN, infliximab; MD, mean difference; RR, risk ratio; SEC, secukinumab.

Safety secondary outcomes

Total adverse events

Table S16. League table of relative effects of interventions on the total adverse events outcome

| **ADA** | . | 0.35 (0.08, 1.65) | . | 0.25 (0.06, 1.14) | 1.07 (0.93, 1.22) | 1.02 (0.82, 1.27) | 1.18 (0.81, 1.71) |
| --- | --- | --- | --- | --- | --- | --- | --- |
| 1.03 (0.78,1.35) | **CER** | . | . | . | 1.03 (0.80, 1.32) | . | . |
| 0.89 (0.33,2.38) | 0.87 (0.32,2.38) | **ETA** | . | 0.71 (0.26, 1.94) | . | . | . |
| 1.02 (0.77,1.35) | 0.99 (0.69,1.41) | 1.14 (0.42,3.14) | **GOL** | . | 1.04 (0.81, 1.34) | . | . |
| 0.80 (0.60,1.07) | 0.77 (0.54,1.12) | 0.89 (0.34,2.32) | 0.78 (0.54,1.13) | **IFX** | 1.28 (0.98, 1.68) | . | . |
| 1.06 (0.94,1.19) | 1.03 (0.80,1.32) | 1.19 (0.45,3.16) | 1.04 (0.81,1.34) | 1.33 (1.02,1.73) | **PBO** | 1.00 (0.90, 1.12) | 0.84 (0.65, 1.08) |
| 1.05 (0.92,1.20) | 1.02 (0.78,1.34) | 1.18 (0.44,3.15) | 1.03 (0.79,1.36) | 1.32 (0.99,1.76) | 0.99 (0.90,1.10) | **SEC** | . |
| 0.95 (0.74,1.22) | 0.93 (0.66,1.30) | 1.07 (0.39,2.92) | 0.94 (0.66,1.32) | 1.20 (0.84,1.70) | 0.90 (0.71,1.14) | 0.90 (0.70,1.16) | **TOF** |

Each cell in the table above contains the risk ratio (RR) and 95% confidence intervals of the intervention in the respective column versus the comparator in the respective row. The Lower triangle presents the result of the network meta-analysis and the upper triangle presents the result of the pairwise meta-analysis. RR greater than 1 favor treatment on the right.

Abbreviations: ADA, adalimumab; ETA, etanercept; GOL, golimumab; IFN, infliximab; SEC, secukinumab; PBO, placebo; TOF, tofacitinib

Figure S18. Network meta-analysis estimates the effect of interventions compared to placebo on the total adverse events

Abbreviations: ADA, adalimumab; CER, certolizumab pegol; ETA, etanercept; GOL, golimumab; IFN, infliximab; SEC, secukinumab; PBO, placebo; TOF, tofacitinib

Figure S19. Ranking of interventions based on their effect on the total adverse events outcome

Abbreviations: ADA, adalimumab; CER, certolizumab pegol; ETA, etanercept; GOL, golimumab; IFN, infliximab; SEC, secukinumab; PBO, placebo; TOF, tofacitinib

Certainty of evidence analysis

Table S17. Certainty of evidence analysis of included studies in network meta-analysis for disease response outcome (ACR 50) at 6-month follow-up

| **Comparison** | **Number of studies** | **Within-study bias** | **Reporting bias** | **Indirectness** | **Imprecision** | **Heterogeneity** | **Incoherence** | **Confidence rating** | **Reason(s) for downgrading** |
| --- | --- | --- | --- | --- | --- | --- | --- | --- | --- |
| Network evidence | | | | | | | | |  |
| **ADA:PBO** | 7 | No concerns | Low risk | No concerns | No concerns | No concerns | No concerns | High | [] |
| **ADA:SEC** | 1 | Major concerns | Low risk | No concerns | Major concerns | No concerns | No concerns | Very Low | ["Within-study bias","Imprecision"] |
| **ADA:TOF** | 1 | No concerns | Low risk | Some concerns | Major concerns | No concerns | No concerns | Very Low | ["Indirectness","Imprecision"] |
| **CER:PBO** | 1 | Some concerns | Low risk | No concerns | No concerns | Some concerns | No concerns | Low | ["Within-study bias","Heterogeneity"] |
| **ETA:PBO** | 3 | No concerns | Low risk | No concerns | No concerns | Some concerns | No concerns | Moderate | ["Heterogeneity"] |
| **GOL:PBO** | 3 | No concerns | Low risk | No concerns | No concerns | No concerns | No concerns | High | [] |
| **IFX:PBO** | 3 | Major concerns | Low risk | Some concerns | No concerns | No concerns | No concerns | Very Low | ["Within-study bias","Indirectness"] |
| **PBO:SEC** | 7 | No concerns | Low risk | No concerns | No concerns | No concerns | No concerns | High | [] |
| **PBO:TOF** | 2 | No concerns | Low risk | Some concerns | No concerns | No concerns | No concerns | Moderate | ["Indirectness"] |
| Indirect evidence | | | | | | | | |  |
| **ADA:CER** | - | Some concerns | Low risk | No concerns | Major concerns | No concerns | No concerns | Very Low | ["Within-study bias","Imprecision"] |
| **ADA:ETA** | - | No concerns | Low risk | No concerns | Major concerns | No concerns | No concerns | Low | ["Imprecision"] |
| **ADA:GOL** | - | No concerns | Low risk | No concerns | Major concerns | No concerns | No concerns | Low | ["Imprecision"] |
| **ADA:IFX** | - | Major concerns | Low risk | Some concerns | Major concerns | No concerns | No concerns | Very Low | ["Within-study bias","Indirectness","Imprecision"] |
| **CER:ETA** | - | Some concerns | Low risk | No concerns | Major concerns | No concerns | No concerns | Very Low | ["Within-study bias","Imprecision"] |
| **CER:GOL** | - | Some concerns | Low risk | No concerns | Major concerns | No concerns | No concerns | Very Low | ["Within-study bias","Imprecision"] |
| **CER:IFX** | - | Some concerns | Low risk | No concerns | Major concerns | No concerns | No concerns | Very Low | ["Within-study bias","Imprecision"] |
| **CER:SEC** | - | Some concerns | Low risk | No concerns | Major concerns | No concerns | No concerns | Very Low | ["Within-study bias","Imprecision"] |
| **CER:TOF** | - | No concerns | Low risk | No concerns | Major concerns | No concerns | No concerns | Low | ["Imprecision"] |
| **ETA:GOL** | - | No concerns | Low risk | No concerns | Major concerns | No concerns | No concerns | Low | ["Imprecision"] |
| **ETA:IFX** | - | Major concerns | Low risk | No concerns | Major concerns | No concerns | No concerns | Very Low | ["Within-study bias","Imprecision"] |
| **ETA:SEC** | - | No concerns | Low risk | No concerns | Major concerns | No concerns | No concerns | Low | ["Imprecision"] |
| **ETA:TOF** | - | No concerns | Low risk | No concerns | Major concerns | No concerns | No concerns | Low | ["Imprecision"] |
| **GOL:IFX** | - | Major concerns | Low risk | No concerns | Major concerns | No concerns | No concerns | Very Low | ["Within-study bias","Imprecision"] |
| **GOL:SEC** | - | No concerns | Low risk | No concerns | Major concerns | No concerns | No concerns | Low | ["Imprecision"] |
| **GOL:TOF** | - | No concerns | Low risk | No concerns | Major concerns | No concerns | No concerns | Low | ["Imprecision"] |
| **IFX:SEC** | - | Major concerns | Low risk | No concerns | Major concerns | No concerns | No concerns | Very Low | ["Within-study bias","Imprecision"] |
| **IFX:TOF** | - | No concerns | Low risk | Some concerns | Major concerns | No concerns | No concerns | Very Low | ["Indirectness","Imprecision"] |
| **SEC:TOF** | - | No concerns | Low risk | No concerns | Major concerns | No concerns | No concerns | Low | ["Imprecision"] |

Table S18. Certainty of evidence analysis of included studies in network meta-analysis for disease response outcome (PsARC) at 6-month follow-up

| **Comparison** | **Number of studies** | **Within-study bias** | **Reporting bias** | **Indirectness** | **Imprecision** | **Heterogeneity** | **Incoherence** | **Confidence rating** | **Reason(s) for downgrading** |
| --- | --- | --- | --- | --- | --- | --- | --- | --- | --- |
| Network evidence | | | | | | | | | |
| **ADA:PBO** | 4 | No concerns | Low risk | No concerns | No concerns | Some concerns | No concerns | Moderate | ["Heterogeneity"] |
| **ADA:TOF** | 1 | No concerns | Low risk | Some concerns | Some concerns | Some concerns | No concerns | Very Low | ["Indirectness","Imprecision", "Heterogeneity"] |
| **CER:PBO** | 1 | Some concerns | Low risk | No concerns | No concerns | Some concerns | No concerns | Low | ["Within-study bias","Heterogeneity"] |
| **ETA:PBO** | 2 | Major concerns | Low risk | No concerns | No concerns | No concerns | No concerns | Low | ["Within-study bias"] |
| **GOL:PBO** | 1 | Major concerns | Low risk | No concerns | Major concerns | No concerns | No concerns | Very Low | ["Within-study bias","Imprecion"] |
| **INF:PBO** | 2 | Some concerns | Low risk | Some concerns | No concerns | No concerns | No concerns | Low | ["Within-study bias", “Indirectness"] |
| **PBO:TOF** | 2 | No concerns | Low risk | Some concerns | No concerns | Major concerns | No concerns | Very Low | ["Indirectness", "Heterogeneity"] |
| Indirect evidence | | | | | | | | | |
| **ADA:CER** | - | Some concerns | Low risk | No concerns | Major concerns | No concerns | No concerns | Very Low | ["Within-study bias","Imprecision"] |
| **ADA:ETA** | - | Major concerns | Low risk | No concerns | Some concerns | Some concerns | No concerns | Very Low | ["Within-study bias", "Imprecision", "Heterogeneity"] |
| **ADA:GOL** | - | Major concerns | Low risk | No concerns | Some concerns | Some concerns | No concerns | Very Low | ["Within-study bias", "Imprecision”, “Heterogeneity"] |
| **ADA:INF** | - | Some concerns | Low risk | Some concerns | Some concerns | Some concerns | No concerns | Very Low | ["Within-study bias", "Indirectness”,"Imprecision","Heterogeneity"] |
| **CER:ETA** | - | Some concerns | Low risk | No concerns | Major concerns | No concerns | No concerns | Very Low | ["Within-study bias","Imprecision"] |
| **CER:GOL** | - | Some concerns | Low risk | No concerns | Some concerns | Some concerns | No concerns | Very Low | ["Within-study bias","Imprecision","Heterogeneity"] |
| **CER:INF** | - | Some concerns | Low risk | No concerns | Major concerns | No concerns | No concerns | Very Low | ["Within-study bias","Imprecision"] |
| **CER:TOF** | - | Some concerns | Low risk | No concerns | Some concerns | Some concerns | No concerns | Very Low | ["Within-study bias","Imprecision","Heterogeneity"] |
| **ETA:GOL** | - | Major concerns | Low risk | No concerns | No concerns | Some concerns | No concerns | Very Low | ["Within-study bias","Heterogeneity"] |
| **ETA:INF** | - | Major concerns | Low risk | No concerns | Major concerns | No concerns | No concerns | Very Low | ["Within-study bias","Imprecision"] |
| **ETA:TOF** | - | No concerns | Low risk | No concerns | No concerns | Some concerns | No concerns | Moderate | ["Heterogeneity"] |
| **GOL:INF** | - | Major concerns | Low risk | No concerns | No concerns | Major concerns | No concerns | Very Low | ["Within-study bias","Heterogeneity"] |
| **GOL:TOF** | - | No concerns | Low risk | No concerns | Major concerns | No concerns | No concerns | Low | ["Imprecision"] |
| **INF:TOF** | - | No concerns | Low risk | Some concerns | Some concerns | Some concerns | No concerns | Very Low | ["Indirectness","Imprecision","Heterogeneity"] |

Table S19. Certainty of evidence analysis of included studies in network meta-analysis for serious adverse events outcome

| **Comparison** | **Number of studies** | **Within-study bias** | **Reporting bias** | **Indirectness** | **Imprecision** | **Heterogeneity** | **Incoherence** | **Confidence rating** | **Reason(s) for downgrading** |
| --- | --- | --- | --- | --- | --- | --- | --- | --- | --- |
| Network evidence | | | | | | | | | |
| **ADA:ETA** | 1 | Major concerns | Low risk | No concerns | Major concerns | No concerns | No concerns | Very Low | ["Within-study bias","Imprecision"] |
| **ADA:IFX** | 1 | Major concerns | Low risk | Some concerns | Major concerns | No concerns | No concerns | Very Low | ["Within-study bias","Indirectness","Imprecision"] |
| **ADA:PBO** | 7 | No concerns | Low risk | No concerns | Major concerns | No concerns | Some concerns | Very Low | ["Imprecision","Incoherence"] |
| **ADA:SEC** | 1 | Major concerns | Low risk | No concerns | Major concerns | No concerns | No concerns | Very Low | ["Within-study bias","Imprecision"] |
| **ADA:TOF** | 1 | No concerns | Low risk | Some concerns | Major concerns | No concerns | No concerns | Very Low | ["Indirectness","Imprecision"] |
| **CER:PBO** | 1 | No concerns | Low risk | No concerns | Major concerns | No concerns | No concerns | Low | ["Imprecision”] |
| **ETA:IFX** | 1 | Major concerns | Low risk | Some concerns | Major concerns | No concerns | No concerns | Very Low | ["Within-study bias","Indirectness","Imprecision"] |
| **ETA:PBO** | 2 | Major concerns | Low risk | No concerns | Major concerns | No concerns | No concerns | Very Low | ["Within-study bias","Imprecision"] |
| **GOL:PBO** | 2 | Some concerns | Low risk | No concerns | Major concerns | No concerns | No concerns | Very Low | ["Within-study bias","Imprecision”] |
| **IFX:PBO** | 2 | Major concerns | Low risk | Some concerns | Major concerns | No concerns | No concerns | Very Low | ["Within-study bias","Indirectness","Imprecision"] |
| **PBO:SEC** | 7 | No concerns | Low risk | No concerns | Some concerns | Some concerns | No concerns | Low | ["Imprecision","Heterogeneity"] |
| **PBO:TOF** | 2 | No concerns | Low risk | Some concerns | Major concerns | No concerns | No concerns | Very Low | ["Indirectness","Imprecision"] |
| Indirect evidence | | | | | | | | | |
| **ADA:CER** | - | No concerns | Low risk | No concerns | Major concerns | No concerns | No concerns | Low | ["Imprecision"] |
| **ADA:GOL** | - | No concerns | Low risk | No concerns | Major concerns | No concerns | No concerns | Low | ["Imprecision"] |
| **CER:ETA** | - | Major concerns | Low risk | No concerns | Major concerns | No concerns | No concerns | Very Low | ["Within-study bias","Imprecision"] |
| **CER:GOL** | - | No concerns | Low risk | No concerns | Major concerns | No concerns | No concerns | Low | ["Imprecision"] |
| **CER:IFX** | - | Major concerns | Low risk | No concerns | Major concerns | No concerns | No concerns | Very Low | ["Within-study bias","Imprecision"] |
| **CER:SEC** | - | No concerns | Low risk | No concerns | Major concerns | No concerns | No concerns | Low | ["Imprecision"] |
| **CER:TOF** | - | No concerns | Low risk | No concerns | Major concerns | No concerns | No concerns | Low | ["Imprecision"] |
| **ETA:GOL** | - | Major concerns | Low risk | No concerns | Major concerns | No concerns | No concerns | Very Low | ["Within-study bias","Imprecision"] |
| **ETA:SEC** | - | Major concerns | Low risk | No concerns | Major concerns | No concerns | No concerns | Very Low | ["Within-study bias","Imprecision"] |
| **ETA:TOF** | - | Major concerns | Low risk | Some concerns | Major concerns | No concerns | No concerns | Very Low | ["Within-study bias","Indirectness","Imprecision"] |
| **GOL:IFX** | - | Major concerns | Low risk | No concerns | Major concerns | No concerns | No concerns | Very Low | ["Within-study bias","Imprecision"] |
| **GOL:SEC** | - | No concerns | Low risk | No concerns | Major concerns | No concerns | No concerns | Low | ["Imprecision"] |
| **GOL:TOF** | - | No concerns | Low risk | No concerns | Major concerns | No concerns | No concerns | Low | ["Imprecision"] |
| **IFX:SEC** | - | Major concerns | Low risk | No concerns | Major concerns | No concerns | No concerns | Very Low | ["Within-study bias","Imprecision"] |
| **IFX:TOF** | - | Major concerns | Low risk | Some concerns | Major concerns | No concerns | No concerns | Very Low | ["Within-study bias","Indirectness","Imprecision"] |
| **SEC:TOF** | - | No concerns | Low risk | No concerns | Major concerns | No concerns | No concerns | Low | ["Imprecision"] |
